# Fine Tuning of the UPR by the Ubiquitin Ligases Siah1/2

**DOI:** 10.1371/journal.pgen.1004348

**Published:** 2014-05-08

**Authors:** Marzia Scortegagna, Hyungsoo Kim, Jian-Liang Li, Hang Yao, Laurence M. Brill, Jaeseok Han, Eric Lau, David Bowtell, Gabriel Haddad, Randal J. Kaufman, Ze'ev A. Ronai

**Affiliations:** 1 Tumor Initiation and Maintenance Program, Cancer Center, Sanford-Burnham Medical Research Institute, La Jolla, California, United States of America; 2 Proteomics Facility, Sanford-Burnham Medical Research Institute, La Jolla, California, United States of America; 3 Department of Pediatrics, University of California, San Diego, La Jolla, California, United States of America; 4 Degenerative Diseases Program, Sanford-Burnham Medical Research Institute, La Jolla, California, United States of America; 5 Department of Biochemistry and Molecular Biology, The University of Melbourne, Parkville, Victoria, Australia; 6 Department of Pathology, The University of Melbourne, Parkville, Victoria, Australia; 7 Sir Peter MacCallum Department of Oncology, The University of Melbourne, Parkville, Victoria, Australia; 8 Cancer Genomics and Genetics, Peter MacCallum Cancer Centre, East Melbourne, Victoria, Australia; University of Mainz, Germany

## Abstract

The endoplasmic reticulum (ER) responds to changes in intracellular homeostasis through activation of the unfolded protein response (UPR). Yet, it is not known how UPR-signaling coordinates adaptation versus cell death. Previous studies suggested that signaling through PERK/ATF4 is required for cell death. We show that high levels of ER stress (i.e., ischemia-like conditions) induce transcription of the ubiquitin ligases Siah1/2 through the UPR transducers PERK/ATF4 and IRE1/sXBP1. In turn, Siah1/2 attenuates proline hydroxylation of ATF4, resulting in its stabilization, thereby augmenting ER stress output. Conversely, ATF4 activation is reduced upon Siah1/2 KD in cultured cells, which attenuates ER stress-induced cell death. Notably, *Siah1a^+/−^::Siah2^−/−^* mice subjected to neuronal ischemia exhibited smaller infarct volume and were protected from ischemia-induced death, compared with the wild type (WT) mice. In all, Siah1/2 constitutes an obligatory fine-tuning mechanism that predisposes cells to death under severe ER stress conditions.

## Author Summary

Maintaining a balanced level of stress (protein folding, reactive oxygen radicals) is important for keeping cellular homeostasis (the ability of a cell to maintain internal equilibrium by adjusting its physiological processes). The accumulation of stress (external or internal) will trigger a well-orchestrated machinery that attempts to restore homeostasis, namely, the unfolded protein response (UPR). The UPR either restores balance to the cells or induces a cell death program, which clears the damaged cell. How this machinery activates cell survival versus cell death is not entirely clear. Here we identify a new layer in the regulation of the UPR, which determines the magnitude of this response. We demonstrate the importance of this newly identified regulatory component for cell death commitments, in response to the more severe conditions (ischemia, lack of oxygen and nutrients). Our findings highlight an undisclosed mechanism that is important for the cell death decision following severe stress conditions, while pointing to the ability to fine tune cellular response to stress.

## Introduction

The endoplasmic reticulum (ER) plays a central role in the folding, assembly, and modification of secretory and cell membrane proteins [1], [2]. Deregulated protein folding affects diverse cellular processes, including transcription, translation, cell cycle, and cell death [3], [4]. The ER responds to exogenous and endogenous stressors that can disrupt protein folding by increasing its protein folding capacity through specialized signaling pathways that are collectively known as the unfolded protein response (UPR) [3]–[6]. The UPR increases transcription of many genes encoding functions in protein folding and secretion, and thus constitutes a coordinated regulatory mechanism that restores protein-folding fidelity in the ER and reestablishes normal cellular homeostasis [1]–[4], [7].

The UPR is coordinated by three main ER-proximal sensors that respond to increased levels of unfolded proteins: ATF6α (activating transcription factor 6), IRE1α (inositol-requiring protein 1), and PERK (PKR-like ER kinase) [3], [4], [7]. ATF6α is proteolytically cleaved upon trafficking to the Golgi to generate the soluble active product, which initiates a transcriptional program to relieve ER stress [8], [9]. IRE1 undergoes autophosphorylation, which activates its intrinsic RNase activity and leads to splicing of XBP1 mRNA to produce the active transcription factor sXBP1 [10], [11]. Activated PERK phosphorylates the eukaryotic initiation factor 2 on the alpha subunit (eIF2α), resulting in an overall attenuation of mRNA translation [12], [13]. Although global protein production is reduced following UPR, the translation of a select group of mRNAs, including the transcription factor ATF4, is increased following PERK activation, via alternative AUG initiation codon selection that occurs when eIF2α is inactivated by phosphorylation [13]–[15].

ATF4 counters the UPR by inducing the expression of genes that improve ER protein folding capacity, facilitate amino acid biosynthesis and transport, and reduce oxidative stress, as well as the pro-apoptotic factor C/EBP homologous protein CHOP [14], [16]–[19].

ER stress response, as part of the UPR, can facilitate the restoration of cellular homeostasis, via the concerted activation of ERAD and the respective transcriptional program (i.e., chaperones) induced by the ATF4, sXPB1 and ATF6α [4], [7]. However, severe ER stress often results in the activation of the cell death program, which is mediated by the UPR transducers CHOP and PUMA [20], [21]. Notably, the mechanism underlying the ability of the UPR to divert cellular survival to death pathways is not well understood. Here we identify the ubiquitin ligases Siah1/2 as important regulatory components in the UPR, which serves as rheostats that can dial up the degree of ER stress response to induce cellular changes that promote cell death. Our data establish the role of Siah1/2 ubiquitin ligases in fine-tuning of the cellular UPR.

Siah1/2 are RING finger ubiquitin ligases that are evolutionarily conserved from *Drosophila melanogaster* to vertebrates [22]. The two human isoforms, *Siah1* and *Siah2*, and the three murine homologues (*Siah1a, Siah1b*, and *Siah2*) are implicated in ubiquitin-dependent degradation of proteins that play key roles in hypoxia and MAPK signaling pathways [23]–[25]. Siah1/2 controls the Ras/Raf signaling pathways and contributes to tumorigenesis by controlling the regulatory protein Sprouty2 [26], [27]. Regulation of the scaffolding protein AKAP121 by Siah2 controls mitochondrial fission under low oxygen conditions, which contributes to ischemia in a mouse myocardial infarction model [28]. Siah1/2 plays an important role in the control of the overall hypoxia response through its effects on the stability of HIF1α, as by affecting the level of HIPK2 (homeodomain-interacting protein kinase 2) and FIH (factor-inhibiting hypoxia-inducible factor), which controls HIF1α own transcription and activity, respectively [29], [30]. HIF1α degradation requires prolyl hydroxylation by the PHD enzymes that enable its recognition and subsequent degradation by pVHL [31], [32]. Whereas PHD2 plays key roles in HIF1α hydroxylation under normoxia, PHD3 and PHD1, which are regulated by Siah2, contribute to the control of HIF1α availability under physiological hypoxic conditions (3–6% O_2_) [25], [33], [34]. Correspondingly, *Siah1/2* mutant mice exhibit phenotypes resembling those seen in HIF1α heterozygous animals [33]. *Siah1/2* deletion in mice attenuates the growth or progression of prostate or melanoma tumors, in a HIF1α-dependent manner [27], [35] and attenuates breast, lung, and pancreatic cancer development [36]–[38]. Genetic inactivation of Siah1/2 also protects from acute myocardial infarction in mice, and affects life span in worms [28]. Mechanisms underlying the regulation of Siah1/2 transcription are confined to Siah1 (but not Siah2), which is transcriptionally induced by p53 following DNA damage [39]–[42]. Here, we identify a mechanism that establishes Siah ubiquitin ligases as integral components of the ER stress response, which fine-tunes the magnitude of UPR, directing cell death programs in response to severe ER stress conditions.

## Results

### UPR induces Siah1/2 transcription via ATF4/sXBP1/ATF6

**Figure 1:**
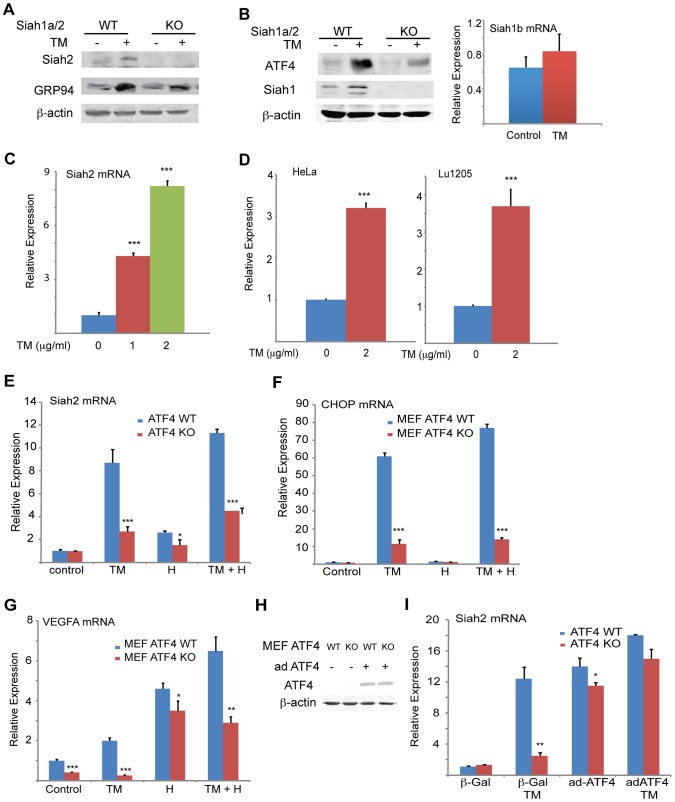
UPR induction of Siah1/2 RNA and protein is ATF4-dependent. (A) TM induces Siah2 protein levels. Litter-matched MEF WT and *Siah1a^−/−^::Siah2^−/−^* cells were treated with TM (1 µg/ml) for 10 h. Whole cell lysates were prepared and analyzed by immunoblotting with the indicated antibodies. (B) TM induces Siah1 protein. Litter-matched MEF WT and *Siah1a^−/−^::Siah2^−/−^* cells were treated with TM (1 µg/ml) for 10 h. Whole cell lysates were prepared and analyzed by immunoblotting with the indicated antibodies. Right panel: ER stress does not induce Siah1b mRNA levels. MEFs from *Siah1a^−/−^::Siah2^−/−^* cells were treated with TM (2 µg/ml) for 6 h and the relative expression of Siah1b mRNA was measured by quantitative real time PCR (qPCR). (C) ER stress induces Siah2 mRNA levels. MEFs from WT animals were treated with the indicated concentrations of TM for 6 h and the relative expression of Siah2 mRNA was measured by qPCR. (D) HeLa and Lu1205 cells were treated with the indicated concentrations of TM for 6 h and the relative expression of Siah2 mRNA was measured by qPCR. (E) ER-stress induction of Siah2 mRNA is attenuated in *Atf4^−/−^* MEFs. Littermate-matched MEFs of the indicated genotypes were subjected to treatment with TM (2 µg/ml), hypoxia (H), or both. RNA prepared 6 h later was used for qPCR to quantify Siah2 transcripts, relative to levels of H3.3A mRNA. (F) CHOP mRNA levels are ATF4 but not hypoxia dependent. MEFs from *Atf4* WT and *Atf4^−/−^* genotypes were subjected to TM or hypoxia or combined treatment and RNA prepared for qPCR analysis of CHOP transcripts. (G) VEGFA mRNA levels are ATF4 dependent under normoxia. Experiment was performed as indicated in panel E, except that qPCR analysis used the VEGFA primers. (H) *Atf4* WT and *Atf4^−/−^* MEFs were infected with ATF4-expressing adenovirus. Cell lysates were prepared and the level of ATF4 protein was detected in immunoblots with the respective antibody. β-actin served as the loading control. (I) Ectopic ATF4 expression rescues Siah2 mRNA levels in TM-treated *Atf4^−/−^* MEFs. MEFs of the indicated genotypes were infected with control (β-Gal) or with ATF4 adenoviruses for 24 h, followed by 6 h exposure to TM (2 µg/ml), as indicated. Relative expression of Siah2 mRNA was quantified by qPCR. *** p<0.0005, ** p<0.005, * p<0.05 compared to non treated (C–D) or to ATF4 WT in the same condition (student's t test). The Western blot experiments were repeated three times and the qPCR results are shown as the means ± S.E. of three independent experiments.

To identify physiological conditions that affect Siah1/2 transcription, we examined cellular responses to key stimuli that are known to influence Siah1/2 substrates or Siah1/2-dependent processes. We considered the UPR as one such condition, given that the Siah1/2 substrates TRAF2, Sprouty2 and PHD3 are implicated in the UPR as part of the ER stress response [43]–[45]. To determine whether conditions that induce UPR also affect Siah2 regulation, we examined Siah2 transcription and protein expression following exposure of cells to the glycosylation inhibitor tunicamycin (TM), which is commonly used to induce UPR. Siah2 protein is barely detectable under normal growth conditions, due to its efficient self-degradation [33], [46]. TM treatment increased Siah2 protein expression in mouse embryo fibroblasts (MEFs) from WT but not *Siah1a^−/−^::Siah2^−/−^* knockout (KO) mice (Figure 1A). Similarly, Siah2 protein expression was increased following treatment of 293T cells with TM (not shown). Since Siah1 is often found to augment Siah2 activity, we also monitored the possible induction of Siah1 by UPR. Siah1 protein expression was found to increase following treatment of MEF cells with TM (Figure 1B). Noteworthy are the levels of ATF4, which appear to correspond to Siah1/2 expression, implying a possible regulatory link between Siah1/2 and ATF4.

**Figure 2:**
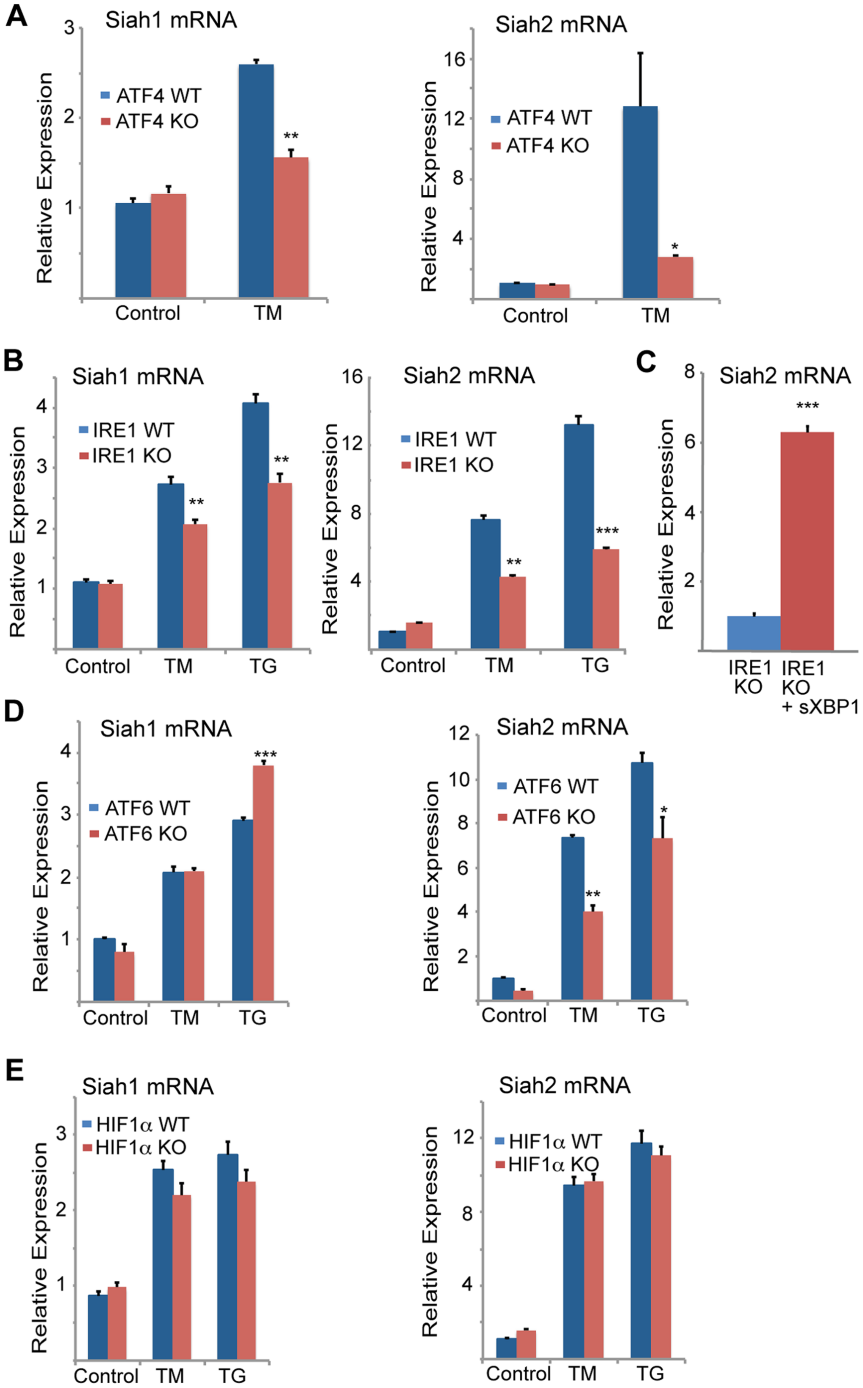
Siah1/2 transcription is induced by ATF4 and sXBP1 upon UPR. (A,B) ER-stress induction of both Siah1 and Siah2 mRNA is attenuated in *Atf4^−/−^* MEFs (A) and *Ire1α^−/−^* MEFs (B). Littermate-matched MEFs of the indicated genotypes were subjected to treatment with TM (2 µg/ml) or TG (1 µM) for 6 h and the relative expression of Siah1 and Siah2 mRNA was measured by qPCR. (C) IRE1α is required for ER-induced Siah2 mRNA levels. Ectopic expression of sXBP1 restores TM-induction of Siah2 mRNA in *Ire1α^−/−^* MEFs. *Ire1α^−/−^* MEFs were infected either with adenovirus encoding either β-gal or sXBP1. After 24 h, RNA was prepared and quantified using qPCR for the relative levels of Siah2 mRNA. (D) ER-stress induction of Siah2 mRNA levels but not of Siah1 mRNA are attenuated in *Atf6α^−/−^* MEFs. Littermate-matched MEFs of the indicated genotypes were subjected to treatment with TM (2 µg/ml) TG (1 µM) for 6 h and the relative expression of Siah1 and Siah2 mRNAs were measured by qPCR. (E) ER Stress induction of Siah1/2 transcripts is not HIF1-dependent. WT and HIF1α KO MEFs were subjected to TM (2 µg/ml) or TG (1 µM) treatment and RNA prepared 6 h later was subjected to analysis of Siah1 or Siah2 transcripts. *** p<0.0005, ** p<0.005, * p<0.07 compared to Ire1 KO (C) or to WT under the same condition (student's t test). The results are shown as the mean values ± S.E. of three independent experiments.

We observed a dose-dependent increase in levels of Siah2 mRNA in both MEFs (>4-fold; Figure 1C) and 293T cells (not shown) following TM treatment. Addition of TM also caused a notable increase in Siah2 mRNA levels in several other cell lines including HeLa, and the melanoma cell line LU1205 (>3-fold; Figure 1D). Collectively, these results indicate that conditions that induce the UPR stimulate Siah2 expression at the RNA and protein levels. Siah1 transcription was also induced following treatment by TM, but to a lower degree (∼2.5-fold) compared with that of Siah2 (∼12-fold, Figure 2A). Of note, murine Siah1 consists of two isoforms (Siah1a and Siah1b), which share 93% homology at the mRNA level. Thus, the ability to distinguish between the two Siah1 murine isoforms, at the protein or RNA levels is limited. To determine whether Siah1a or Siah1b (or both) are induced following TM treatment, MEFs from *Siah1a^−/−^::Siah2^−/−^* KO mice (where Siah1a gene is deleted) were treated with TM. Addition of TM did not cause any induction of Siah1 in *Siah1a^−/−^::Siah2^−/−^* KO cells, implying that Siah1b transcription is not induced after TM treatment (Figure 1B). We therefore concluded that the changes we monitor in the WT MEFs are likely to be of the Siah1a isoform (data shown below, Figure 2A).

To explore the newly identified link between the UPR and Siah2 expression we asked whether Siah2 transcription was regulated by transducers of the UPR. ATF4 is one of several key UPR transcriptional activators [14], [47], [48], suggesting it may play a role in Siah2 transcription. Indeed, we found that TM-stimulated Siah2 transcription was reduced from 8-fold in WT MEFs to ∼2.2-fold in MEFs from *Atf4^−/−^* mice (Figure 1E). These findings provide genetic evidence for a role of ATF4 in the regulation of Siah2 transcription in response to UPR. Hypoxia, which was previously shown to stimulate Siah2 transcription [33], elicited a less pronounced effect than TM on Siah2 transcription (2.2-fold vs. 8-fold; Figure 1E). Corresponding changes in ATF4 transcriptional targets, CHOP and VEGFA were observed under these conditions (Figures 1F,G).

To confirm that ATF4 is critical for Siah2 transcription in response to UPR, we re-expressed ATF4 in *Atf4^−/−^* cells. Indeed, forced expression of ATF4 (Figure 1H) enhanced induction of Siah2 transcription following treatment with TM (Figure 1I), suggesting that ATF4 is required for induction of Siah2 transcription following UPR stimuli, mediated here by TM.

To determine the degree of ATF4-dependent activation of Siah1 and Siah2 following UPR stimuli, MEFs from ATF4 WT and KO genotypes were subjected to TM treatment. TM induced Siah2 mRNA to a greater degree than Siah1, and ATF4^−/−^ MEFs exhibited a more pronounced reduction of Siah2 transcription (Figure 2A). These data indicate that the Siah1 and Siah2 genes are transcriptionally induced by the UPR through ATF4, with Siah2 being the primary responder to the UPR.

We next determined whether other major UPR transducers, IRE1α/sXBP1 and ATF6α, another key UPR transcription factor [49], play a role in the activation of Siah transcription. Whereas TM- and more effectively thapsigargin (TG)-induced Siah2 transcription to a greater degree (8- and ∼13-fold, respectively) than Siah1 transcription (∼2.7–5 and ∼4-fold, respectively), in *Ire1α^−/−^* MEFs Siah2 activation was attenuated to a greater degree, compared with Siah1 (Figure 2B). Notably, re-expression of sXBP1 in *Ire1α* ^−/−^ cells effectively rescued expression of Siah2 (Figure 2C). Similar analysis in *Atf6α^−/−^* MEFs revealed a role of ATF6α in Siah2 transcription (Figure 2D, right panel), albeit lower than was observed for ATF4 and IRE1α. In contrast, ATF6α did not have an effect on Siah1 transcription (Figure 2D, left panel). Collectively these data suggest that the UPR induced Siah2 transcription to a greater degree than the transcription of Siah1, and that among the UPR sensors, ATF4 is the most potent mediator of this activation, followed by sXBP1, and to lesser degree ATF6α.

Since hypoxia was also found to affect Siah2 transcription, albeit to lower degree, we assessed whether the activation of Siah1/2 transcription upon UPR may be mediated by HIF1α. To this end we assessed the degree of TM/TG activation of Siah1/2 in WT versus HIF1α^−/−^ MEFs. Notably, the lack of HIF1α did not elicit a marked effect on the level of Siah1/2 transcriptional activation in response to UPR stimuli (Figure 2E), suggesting that the induction of Siah1/2 transcription under these conditions is HIF1α-independent.

**Figure 3:**
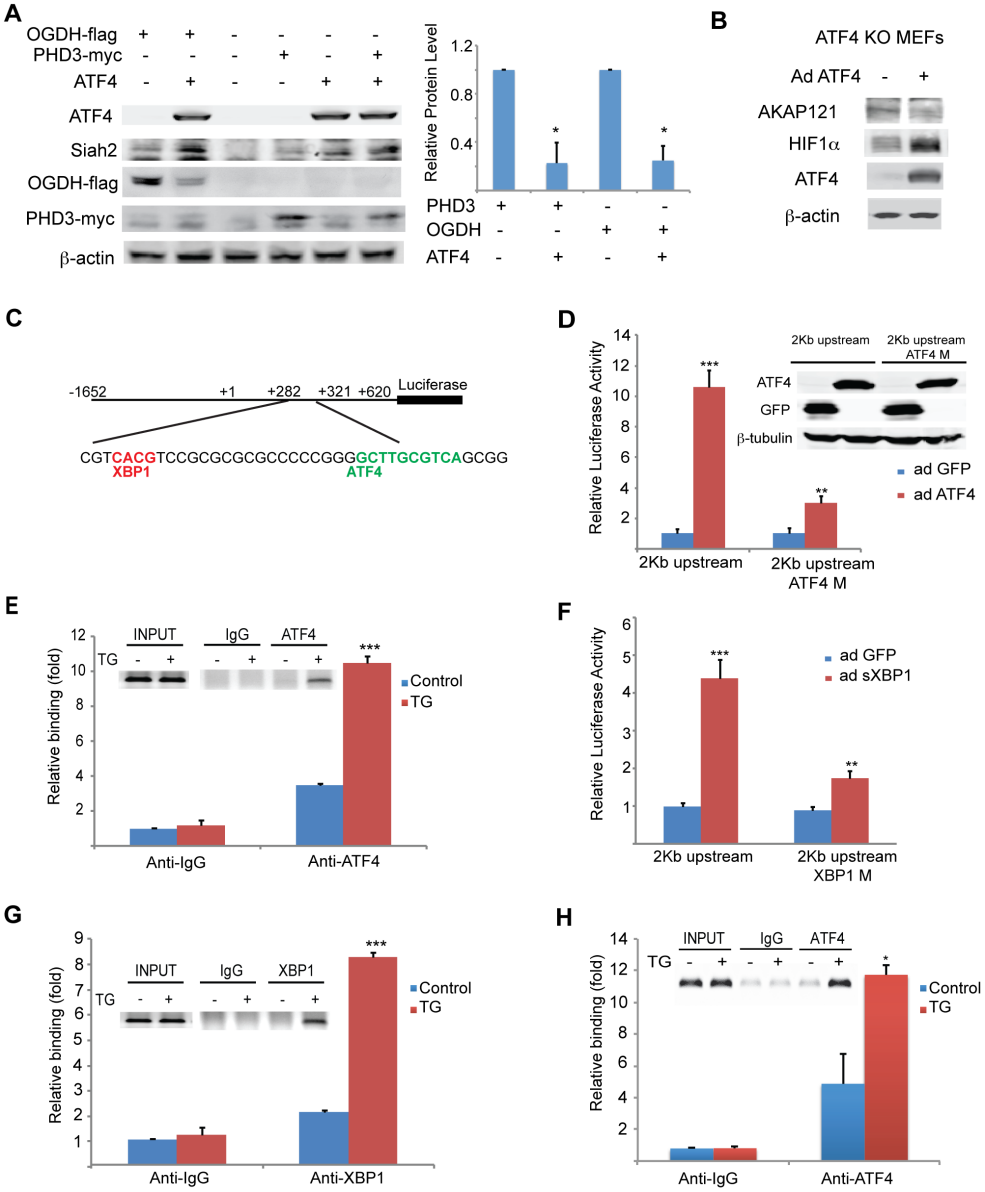
Mapping ATF4 and sXBP1 response elements on Siah1a/2 regulatory regions. (A) Overexpression of ATF4 decreases ectopic PHD3 and OGDH protein levels. 293T cells were transfected with myc-tagged PHD3 or flag-tagged OGDH, and 12 h later were infected with ATF4 virus for 24 h. Whole cell lysates were analyzed by Western blotting with the indicated antibodies. Three independent experiments were carried out and Western blot bands corresponding to PHD3 and OGDH were quantified and normalized against β-actin (graph on the right). (B) Overexpression of ATF4 decreases AKAP121 and increases HIF-1α protein level. *Atf4^−/−^* MEFs were infected with ATF4 virus for 24 h and then exposed to hypoxia (1% O_2_) for 8 h. Whole cell lysates were analyzed by Western blotting with the indicated antibodies. (C) Schematic diagram of the human Siah2 promoter, showing the sequence of ATF4 and sXBP1 response elements. The transcription start site is marked as +1. (D) Mapping ATF4 response element on Siah2 promoter. A 2 Kb fragment containing the upstream and the 5′-UTR domains were cloned into luciferase constructs, in which the putative ATF4 binding site (ATF4M; +308 bp) within the 5′-UTR region was mutated. Siah2 promoter activity was monitored in MEFs infected with either GFP or ATF4 expressing adenovirus and 24 h later cells were harvested and proteins were used to perform luciferase assays and to perform Western blotting with the indicated antibodies. (E) ChIP confirms ATF4 occupancy of corresponding Siah2 promoter site. MEFs were exposed to TG for 5 h and ChIP analysis was performed using antibodies to ATF4 or control IgG as indicated. A set of primers was used to quantify a 214 bp fragment containing the ATF4 site in position +308 using qPCR. (F) Mapping the XBP1 response element in the Siah2 promoter. A 2 Kb fragment containing the upstream and the 5′-UTR domains was cloned into luciferase constructs, in which the putative sXBP1 binding site (XBP1M; +287) within the 5′-UTR region was mutated. Siah2 promoter activity was monitored in MEFs infected with adenoviruses expressing either GFP or sXBP1. After 24 h, cells were harvested and proteins were used to perform luciferase assays. (G) ChIP confirms XBP1 occupancy of corresponding Siah2 promoter sites. MEFs were exposed to TG for 5 h and ChIP analysis was performed using antibodies to XBP1 or control IgG as indicated. A set of primers was used to quantify a 214-bp fragment containing the Xbp1 site in position +287 using qPCR. (H) Mapping the ATF4 response element in the Siah1a promoter. ChIP analysis confirms ATF4 occupancy of corresponding Siah1a intron site. MEFs were exposed to TG for 5 h and ChIP analysis was performed using antibodies to ATF4 or control IgG as indicated. A set of primers was used to quantify a 154-bp fragment containing the ATF4 site in position +4,710 using qPCR. *** p<0.0005, ** p<0.005, * p<0.05 compared to ad GFP (D, F) or to control in the same condition (student's t test). The Western blot experiments were repeated three times and the qPCR results are shown as the mean values ± S.E. of three independent experiments.

We next asked whether the effect of ATF4 on Siah2 transcription was reflected in Siah2 ubiquitin ligase activity. Overexpression of ATF4 increased the protein levels Siah2, with concomitant decrease in the protein levels PHD3 and OGDH, representing Siah2 substrates (Figure 3A). In agreement, re-expression of ATF4 in *Atf4^−/−^* cells that were maintained under hypoxic conditions increased expression levels of HIF1α (PHD3 substrate regulated by Siah2) while reducing the expression of a Siah2 substrate AKAP121 (Figure 3B), consistent with increased Siah2 availability (Figure 3B). This finding supports a role for the UPR-induced transcription factor ATF4 in the regulation of Siah2 ubiquitin ligase activity.

The finding that PERK/ATF4 and IRE1α/sXBP1 increase the level of Siah1/2 mRNA prompted us to search for ATF4 and sXBP1 response elements within the Siah2 promoter region. To this end, we cloned a 2 kb fragment (position −1652; +620) including a region upstream of the start site and the 5′UTR, and two intronic regions into a luciferase reporter construct (Figure 3C). The luciferase activity driven by the 2 kb promoter region containing the 5′-UTR revealed a marked (10-fold) ATF4-dependent increase in (Figure 3D). Within this region we mapped two putative ATF4 response elements (TTxCATCA; Figure 3C), which were then mutated individually. Mutation of only one of the two elements, which are conserved in human and mouse (+308 relative to the transcription start position; ATF4 M), resulted in a marked decrease in ATF4-dependent luciferase activity (Figure 3D). Chromatin immuneprecipitation (ChIP) experiments confirmed that ATF4 bound directly to the Siah2 promoter element within 308–317 following TG treatment (Figure 3E). Together, these data establish that expression of Siah2 mRNA is regulated by ATF4, and that such regulation plays an important role in upregulating Siah2 expression in response to ER stress.

The Siah2 promoter also contains two putative HIF1α response elements (HRE) that can be occupied by HIF1α, which overlaps the sXBP1 binding site (Figure 3C). To map putative sXBP1 sites, we monitored luciferase activity driven by Siah2 promoter, which was mutated on the primary putative sXBP/HRE sites. Luciferase activities identified the sXBP1 response element at position +287, but not the one at position +272, as the site responsible for the activation of Siah2 transcription (Figure 3F). Mutation of the 287 site attenuated sXBP1-dependent activation of Siah2 promoter-driven luciferase activity (Figure 3F). Finally, ChIP experiments confirmed that sXBP1 bound to Siah2 promoter following treatment of cells with TG (Figures 3C,G). These data establish the regulation of Siah2 mRNA levels by two of the three UPR signaling sensors—PERK/ATF4 and IRE1α/sXBP1. Notably, ATF4 and sXBP1 occupy distinct sites within the Siah2 promoter. Similar analysis, performed for the Siah1a promoter, identified ATF4 binding within the first intron of Siah1a (+4710, Figure 3H), consistent with previous ChIP-Seq reports [19]. These findings demonstrate the regulation of Siah1a/2 transcription by the UPR transducers ATF4 and sXBP1.

### Siah1/2-dependent gene expression analysis confirms an ER stress signature

Gene expression profiling was performed to identify Siah1/2-dependent changes in cells subjected to ER stress, hypoxia (1% O_2_), and combined glucose/oxygen deprivation. To this end, we compared gene expression profiles of WT and *Siah1a^−/−^::Siah2^−/−^* MEFs that were subjected to TM, TG, hypoxia (oxygen deprivation), glucose deprivation, or glucose+oxygen deprivation.

**Figure 4:**
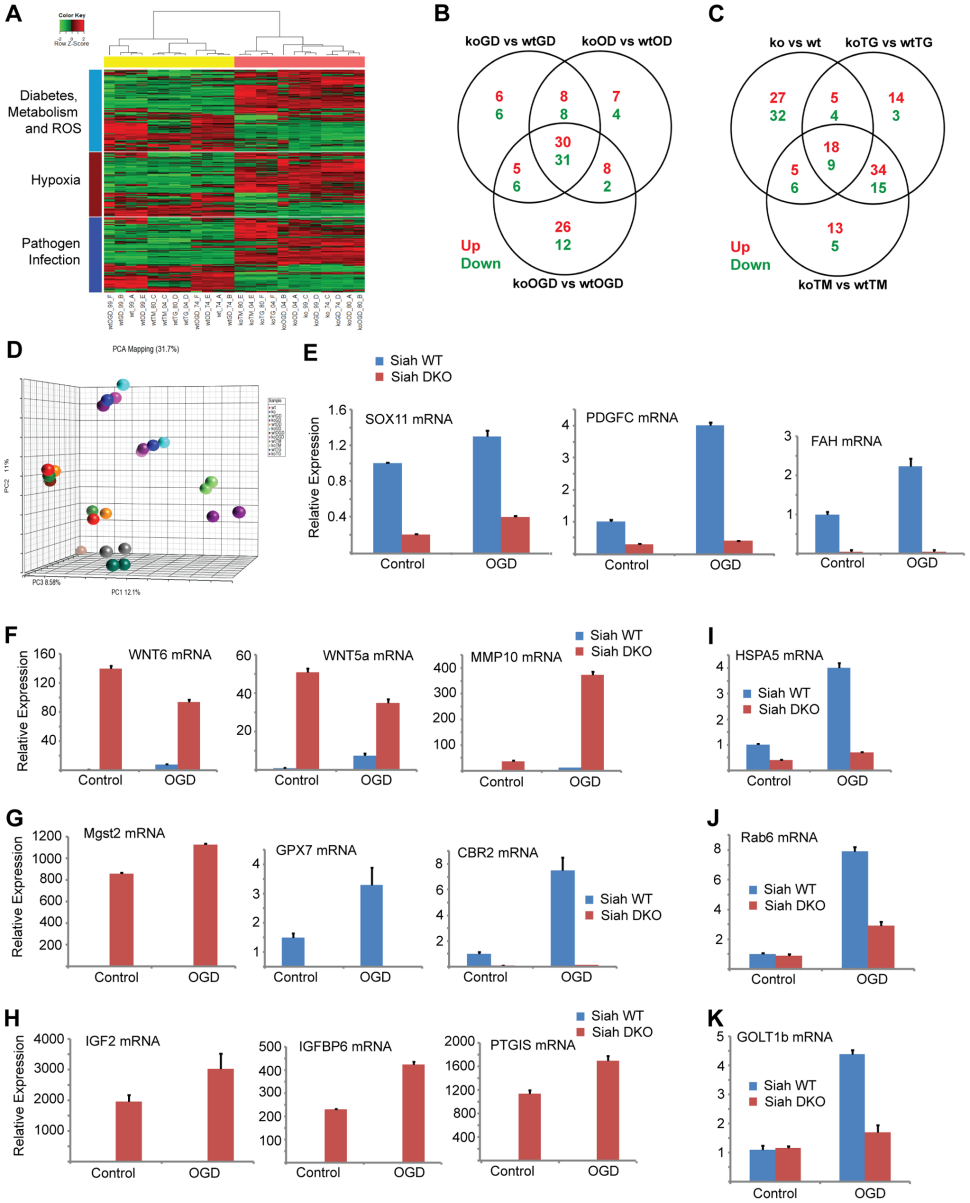
Siah1/2-dependent gene expression analysis confirms an ER stress signature. (A) Heat map showing genes involved in diabetes, metabolism and ROS functions (light blue bar), hypoxia and related signaling (brown bar), and pathogen infection and related signaling (blue bar). Each bar represents a block of significant genes, which is involved in the same functional group. The selected genes have p-values less than or equal to 0.05 and fold-changes are greater than or equal to 2 (both directions). In the heatmap, the normalized expression signals are shown from green to red (lower signal to higher signal). The bars on the left of the heatmap indicate the functional groups. Each bar represents a block of significant genes, which involved in the same functional group. (B–C) Venn diagram representing the overlaps of significantly down- (green), and up- (red) expressed genes involved in the functional groups as indicated in the heatmap. Panel B shows genes from pairwise comparisons: knock out glucose deprivation (koGD) versus wtGD, ko oxygen deprivation (koOD) versus wtOD and ko oxygen and glucose deprivation (koOGD) versus wtOGD. Panel c shows genes from pairwise comparisons: ko versus wt, koTG versus wtTG and koTM versus wtTM. (D) Principal component analysis (PCA) 3D plot for the microarray data set. Each spot represents an individual array, and is colored based on treatment group. (E–H) Validation of representative genes from each of the main pathways identified to be Siah1/2-dependent. Siah WT and *Siah1a^+/−^::Siah2^−/−^* (double knock out; DKO) MEF cells were subjected to OGD for 12 h. The relative transcription level was determined by qPCR. Shown are representative genes of the hypoxia (panel E and F), and diabetes/metabolism (panel G and H) pathways found to exhibit increase or decrease expression upon KO of Siah1a/2. The results are shown as the mean values ± S.E. of three independent experiments. (I–K) RNA prepared from Siah1a/2 WT and Siah DKO MEF cells was used for qPCR analysis. The expression of the transcripts from HSPA5 (I), Rab6 (J), GOLT1b (K) were validated by real time qPCR analysis. The results are shown as the mean values ± S.E. of three independent experiments.

When comparing all six experimental conditions using Ingenuity Pathway Analysis (IPA; canonical pathway analysis), the most significant changes in gene expression between WT and *Siah1a^−/−^::Siah2^−/−^* expressing cells were in the HIF1α/hypoxia signaling pathway (Figure 4A), consistent with our earlier studies showing that Siah2 affects this pathway through its regulation of PHD1/3 stability [33]. Significantly, this analysis also supports the role of Siah1/2 in the regulation of genes implicated in the ER stress response. As shown in the general heat map, major clusters identified to be Siah1/2-dependent included diabetes and metabolism, in addition to hypoxia signaling. Notably, clusters associated with pathogen infection, which were previously associated with the ER stress response [50]–[52], were also identified (Figure 4A; Table 1). The Venn diagram (Figures 4B,C) represents the overlaps of significantly down- (green), and up- (red) regulated genes (listed in Tables 2–3) involved in the functional groups as indicated in the heatmap. A principal component analysis (PCA) for the microarray data set confirmed the clustering of the individual arrays based on their treatment groups (Figure 4D).

**Table 1:** List of top up-regulated (at least 10-fold change, 10 genes) and down-regulated (at least 5-fold change, 11 genes) across all comparisons.

Symbol	Gene Name	KO vs WT	koOD vs wtOD	koGD vs wtGD	koOGD vs wtOGD	koTG vs wtTG	koTM vs wtTM
*Gpr149*	G protein-coupled receptor 149	−38.08	−39.84	−33.06	−17.30	−8.31	−8.38
*Fah*	fumarylacetoacetate hydrolase (fumarylacetoacetase)	−13.75	−15.92	−11.80	−12.20	−14.00	−16.27
*Slc35g2*	solute carrier family 35, member G2	−13.14	−11.61	−10.46	−17.33	−13.03	−13.25
*Foxs1*	forkhead box S1	−13.13	−12.96	−9.95	−8.98	−5.41	−10.27
*Cldn1*	claudin 1	−12.48	−12.17	−17.28	−5.27	−6.93	−8.40
*Thbd*	thrombomodulin	−9.65	−10.18	−10.87	−27.78	−13.74	−7.84
*Gpx7*	glutathione peroxidase 7	−8.09	−7.44	−8.11	−7.76	−6.31	−6.76
*Xlr4a*	X-linked lymphocyte-regulated 4A	−6.93	−6.97	−7.99	−8.65	−26.12	−20.75
*Fam110c*	family with sequence similarity 110, member C	−6.04	−7.48	−6.59	−10.37	−19.78	−17.99
*Gap43*	growth associated protein 43	−5.81	−6.46	−5.63	−10.34	−12.15	−14.59
*Siah1a*	siah E3 ubiquitin protein ligase 1	−5.08	−5.68	−5.39	−5.58	−7.47	−8.54
*Stxbp2*	syntaxin binding protein 2	10.55	11.19	11.58	13.73	12.13	10.83
*Prss53*	protease, serine, 53	13.72	14.89	15.33	16.99	10.00	17.73
*Gas7*	growth arrest specific 7	17.74	20.68	19.16	17.84	11.82	24.30
*Igfbp6*	insulin-like growth factor binding protein 6	36.66	37.87	47.60	33.41	28.42	31.10
*Prl2c2*	prolactin family 2, subfamily c, member 2	51.80	76.06	50.25	42.81	42.64	38.11
*Prl2c3*	prolactin family 2, subfamily c, member 3	59.47	79.07	57.08	48.44	52.93	39.07
*H19*	H19, imprinted maternally expressed transcript (non-protein coding)	63.91	67.84	71.70	68.69	116.89	206.50
*Prl2c4*	prolactin family 2, subfamily c, member 4	65.30	96.14	57.96	42.19	40.42	39.18
*Ptgis*	prostaglandin I2 (prostacyclin) synthase	116.48	95.67	113.69	124.07	75.85	91.20
*Igf2*	insulin-like growth factor 2 (somatomedin A)	224.57	239.35	241.19	191.34	173.65	230.40

**Table 2:** List of common significant differential expression genes among KO versus WT, koTG versus wtTG, and koTM versus wtTM (Figure 4b Venn diagram).

Gene Symbol	KO vs WT	koTG vs wtTG	koTM vs wtTM	Functional Group
*Sema7a*	−7.54	−3.03	−5.03	hypoxia
*Ccl5*	−7.09	−4.21	−2.97	diabetes, metabolism, ROS and pathogen infection
*Siah1a*	−5.08	−7.47	−8.54	pathogen infection
*Sox11*	−4.94	−4.11	−5.55	hypoxia
*Sox2*	−3.35	−3.08	−2.8	hypoxia
*H2-DMb1*	−3.12	−2.29	−2.39	diabetes, metabolism, ROS and pathogen infection
*Ccnd2*	−2.58	−2.94	−3.77	diabetes, metabolism, ROS and hypoxia
*Hspb2*	−2.5	−4.32	−3.45	hypoxia
*Rhoc*	−2.26	−3.29	−2.37	pathogen infection
*Fzd1*	2.03	3.69	6.11	hypoxia, and pathogen infection
*Fzd6*	2.05	2.83	2.37	hypoxia, and pathogen infection
*Tgm2*	2.46	3.81	7.32	hypoxia
*Aldh3b1*	2.51	4.12	3.36	diabetes, metabolism, ROS, hypoxia, and pathogen infection
*Wnt6*	2.55	4.68	4.77	hypoxia, and pathogen infection
*Col3a1*	2.85	3.38	5.08	diabetes, metabolism, and ROS
*Ednra*	3.04	2.5	4.86	diabetes, metabolism, and ROS
*Pik3r3*	3.06	4.56	4.62	diabetes, metabolism, ROS, hypoxia, and pathogen infection
*Pdgfra*	3.34	8.43	24.71	diabetes, metabolism, and ROS
*Cxcl12*	3.51	10.77	21.3	hypoxia, and pathogen infection
*Ror2*	3.75	3.11	2.9	pathogen infection
*Col1a1*	3.93	3.82	5.2	diabetes, metabolism, ROS and pathogen infection
*Mapk12*	4.49	4.36	3.73	hypoxia
*Wnt5a*	5.41	3.76	3.89	hypoxia, and pathogen infection
*Sox12*	5.47	4.76	6.16	hypoxia
*Tnfrsf11b*	5.71	4.88	2.77	diabetes, metabolism, ROS and pathogen infection
*Mmp3*	5.86	2.7	4.96	hypoxia, and pathogen infection
*Cdh3*	6.13	5.76	5.67	hypoxia

**Table 3:** List of common significant differential expression genes among KO versus WT, koOD versus wtOD, and koOGD versus wtOGD (Figure 4c Venn diagram).

Gene Symbol	KO vs WT	koOD vs wtOD	koOGD vs wtOGD	Functional Group
*Thbd*	−9.65	−10.18	−27.78	diabetes, metabolism, ROS
*H2-D1*	−8.11	−7.82	−7.62	diabetes, metabolism, ROS and pathogen infection
*Gpx7*	−8.09	−7.44	−7.76	diabetes, metabolism, ROS
*Sema7a*	−7.54	−7.3	−4	hypoxia
*Ccl5*	−7.09	−7.32	−7.14	diabetes, metabolism, ROS and pathogen infection
*H2-Q2*	−6.67	−4.69	−5.5	diabetes, metabolism, ROS and pathogen infection
*Cbr2*	−5.65	−8.21	−10.4	diabetes, metabolism, ROS
*Psmb9*	−5.61	−7.75	−10.68	pathogen infection
*Psmb8*	−5.34	−4.68	−5.13	diabetes, metabolism, ROS and pathogen infection
*Gm8909*	−5.29	−5.55	−5.25	diabetes, metabolism, ROS and pathogen infection
*H2-D1*	−5.13	−6.73	−7.21	diabetes, metabolism, ROS and pathogen infection
*Siah1a*	−5.08	−5.68	−5.58	pathogen infection
*Sox11*	−4.94	−5.93	−3.79	hypoxia
*Cyp2c55*	−4.35	−4.16	−6.94	diabetes, metabolism, ROS
*Traf1*	−3.93	−3.74	−3.04	pathogen infection
*Efna1*	−3.82	−4.84	−7.09	hypoxia
*Oas1g*	−3.68	−3.13	−2.63	diabetes, metabolism, ROS and pathogen infection
*Igfbp2*	−3.6	−2.81	−8.22	diabetes, metabolism, ROS
*Tlr2*	−3.4	−3.01	−3.61	diabetes, metabolism, ROS and pathogen infection
*Sox2*	−3.35	−3.56	−3.52	hypoxia
*H2-T10*	−3.27	−2.74	−3.11	diabetes, metabolism, ROS and pathogen infection
*H2-DMb1*	−3.12	−2.95	−2.63	diabetes, metabolism, ROS and pathogen infection
*Ifngr2*	−3.11	−2.3	−2.42	diabetes, metabolism, ROS and pathogen infection
*Tap1*	−2.53	−2.35	−2.91	diabetes, metabolism, ROS and pathogen infection
*Sema4f*	−2.5	−3.42	−2.65	hypoxia
*Mmp16*	−2.36	−2.25	−2.83	hypoxia
*Ptges*	−2.35	−5.08	−6.49	diabetes, metabolism, ROS
*Prkca*	−2.32	−2.11	−2.72	diabetes, metabolism, ROS, hypoxia and pathogen infection
*Tap2*	−2.21	−2.01	−3.01	pathogen infection
*Pdgfc*	−2.15	−2.25	−2.11	diabetes, metabolism, ROS, hypoxia and pathogen infection
*Gstm5*	−2.1	−2.21	−2.36	diabetes, metabolism, ROS, hypoxia and pathogen infection
*Nfkbia*	−2.02	−2.48	−2.83	diabetes, metabolism, ROS and pathogen infection
*Fzd1*	2.03	2.81	2.38	hypoxia and pathogen infection
*Srgap3*	2.1	2.17	2.66	hypoxia
*Chst15*	2.21	2.2	2.19	pathogen infection
*Wnt7b*	2.25	3.58	3.24	hypoxia and pathogen infection
*Adam23*	2.31	2.16	2.79	hypoxia
*Ccl7*	2.4	2.2	3.59	pathogen infection
*Aldh3b1*	2.51	2.73	2.1	diabetes, metabolism, ROS, hypoxia and pathogen infection
*Plcg2*	2.52	2.09	2.36	pathogen infection
*Smox*	2.59	2.72	2.76	diabetes, metabolism, ROS and pathogen infection
*Myl7*	2.72	2.27	5.34	diabetes, metabolism, ROS and hypoxia
*Socs2*	2.88	2.12	4.8	diabetes, metabolism, ROS
*Slc2a3*	3.08	7.59	20.75	hypoxia
*Pla2g7*	3.19	4.26	3.08	diabetes, metabolism, ROS and pathogen infection
*Gstk1*	3.2	2.95	2.83	diabetes, metabolism, ROS, hypoxia and pathogen infection
*Gsto2*	3.21	3.18	6.84	diabetes, metabolism, ROS, hypoxia and pathogen infection
*Dkk3*	3.42	3.24	4.28	hypoxia and pathogen infection
*Mgst2*	3.44	3.07	3.81	diabetes, metabolism, ROS, hypoxia and pathogen infection
*Cxcl12*	3.51	2.94	3.72	hypoxia and pathogen infection
*Ror2*	3.75	3.59	3.47	pathogen infection
*Gstt3*	3.85	3.47	4.01	diabetes, metabolism, ROS
*Mapk12*	4.49	3.6	3.32	hypoxia
*Adam23*	4.82	3.79	3.25	hypoxia
*Wnt5a*	5.41	5.25	4.53	hypoxia and pathogen infection
*Sox12*	5.47	5.64	3.47	hypoxia
*Tnfrsf11b*	5.71	4.22	5.88	diabetes, metabolism, ROS and pathogen infection
*Mmp3*	5.86	5.49	8.16	hypoxia and pathogen infection
*Cdh3*	6.13	5.15	5.85	hypoxia
*Hs3st1*	7.92	9.27	7.1	pathogen infection
*Mmp10*	8.65	7.95	15.42	hypoxia
*Ptx3*	10.76	11.96	8.42	pathogen infection
*Grb10*	19.95	25.65	16.47	diabetes, metabolism, ROS
*Igfbp6*	36.66	37.87	33.41	diabetes, metabolism, ROS
*Ptgis*	116.48	95.67	124.07	diabetes, metabolism, ROS

Approximately 35 genes were up- or down-regulated between the hypoxia (OD), hypoxia and glucose deprivation (OGD) and control groups, among the WT and *Siah1a^−/−^::Siah2^−/−^* cells. Those genes were primarily clustered within the hypoxia and diabetes signaling pathways. Somewhat similarly, the comparison among the WT and *Siah1a^−/−^::Siah2^−/−^* groups subjected to TG or TM versus control identified 27 up-regulated and 13 down-regulated genes, which primarily clustered within the cellular movement and development functional groups. qPCR analysis of representative genes from each of the major three clusters, confirmed the changes predicted by the expression array (Figures 4E–H).

We next compared the expression of genes that were significantly altered in a Siah2- dependent manner with previously reported datasets for ATF4-, PERK-, and HIF1α-dependent gene expression. Gene Set Enrichment Analyses (GSEA) confirmed the effect of Siah1/2 on expression of genes associated with ER stress, metabolic signaling and ER-Golgi transport (Table 4). Representative genes from each of these clusters were confirmed by qPCR (Figures 4I–K). Taken together, this analysis confirmed changes associated with ER stress that had not previously been associated with Siah1/2 signaling, substantiating Siah1/2 as important coordinator of ER stress through the ATF4 and sXbp1 pathways.

**Table 4:** Representative of ER stress genes deregulated in both the microarray from Siah1a/2 KO MEF cells and the published microarray performed on ATF4 KO MEF cells or PERK KO liver after TM treatment.

Gene Symbol	Gene description	KO vs WT TM	KO vs WT OGD
HSPA5	heat shock protein 5 (GRP78)	−2	−2.5
GDF15	growth differentiation factor 15	−3.8	−2.4
STARD5	StAR-related lipid transfer	−2.8	−2.4
CHAC1	ChaC, cation transport regulator-like 1	−1	−2.2
PDIA6	protein disulfide isomerase associated 6	−2.4	−1.95
STARD5	StAR-related lipid transfer	−2.8	−2.4
GJB2	gap junction protein, beta 2	−4.7	−4
BTC	betacellulin, epidermal growth factor family member	−2.8	−3
ARF6	ADP-ribosylation factor 6	−1.85	−1.3
FGF21	fibroblast growth factor 21	−2.2	−5
DGAT2	diacylglycerol O-acyltransferase 2	−2.2	−1.2
PRKG2	protein kinase, cGMP-dependent, type II	−2	-
RASSF5	Ras association (RalGDS/AF-6) domain family 5	−2.1	−1.4
AREG	amphiregulin	−6.5	-
PHGDH	3-phosphoglycerate dehydrogenase	−1.8	−1
PTPN2	protein tyrosine phosphatase, non-receptor type 2	−1.3	−2
GDF15	growth differentiation factor 15	−3.8	−5
STARD5	StAR-related lipid transfer	−2.8	−2.4
IGF2	insulin-like growth factor 2	230	191
GOLT1b	Golgi transport 1 homolog B	−1.5	−1.5
sec22b	SEC22 vesicle trafficking protein homolog B	−1	−1.6
rab6	member RAS oncogene family	−1.6	−2

### Siah1/2 activation by the UPR augments ATF4 expression through inactivation of PHD1/3

**Figure 5:**
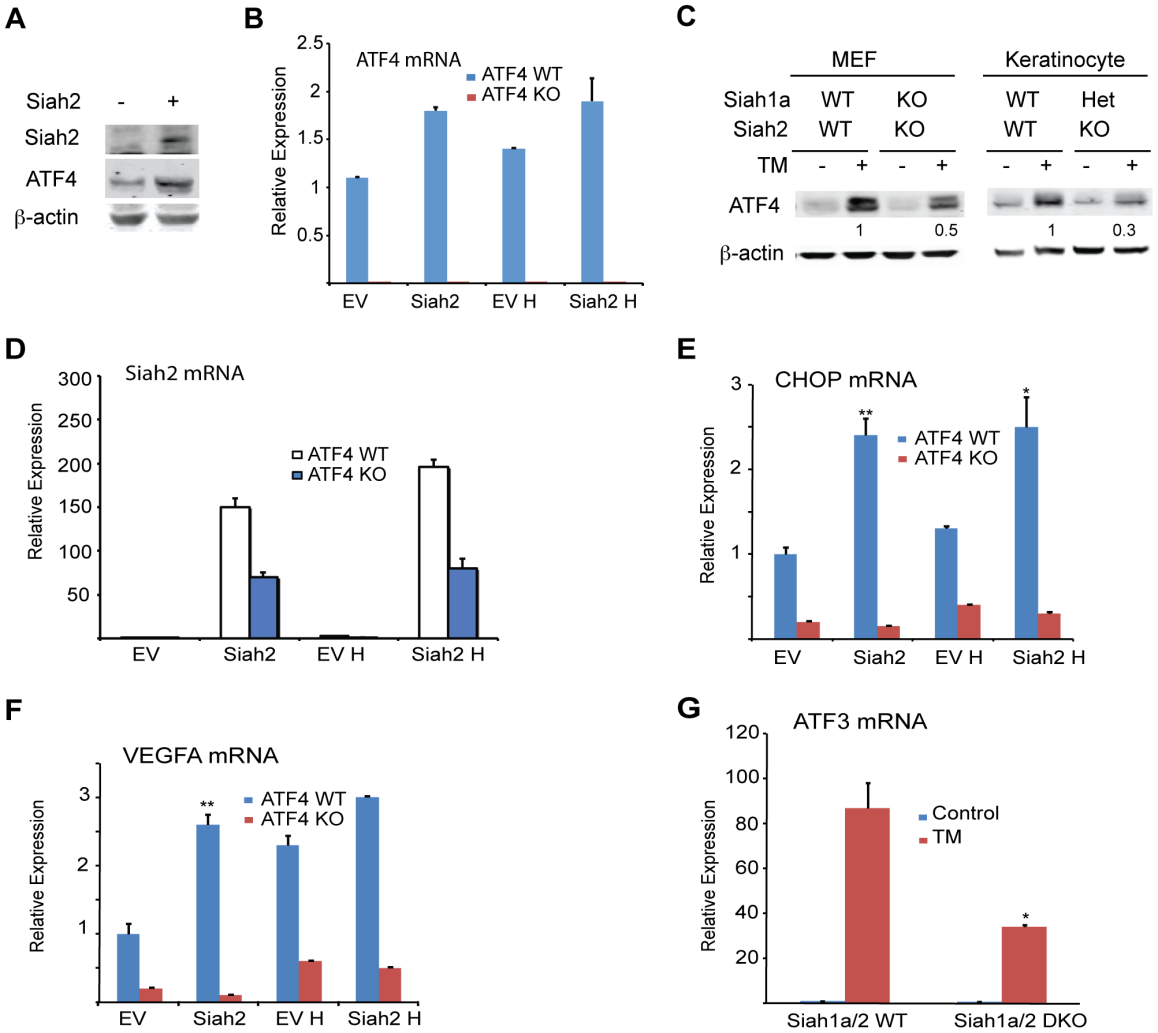
ATF4 transcriptional activities during UPR are Siah1/2-dependent. (A) Siah2 expression augments ATF4 protein levels. WT MEFs were transfected either with control or Siah2 expression vectors, and 24 h later the cells were collected. Harvested proteins were analyzed for ATF4, Siah2 and β-actin. (B) WT and *Atf4^−/−^* MEFs were transfected either with control or Siah2-expressing vectors and after 24 h were subjected to hypoxia (H; 1% O_2_; 6 h). Expression of ATF4 mRNA was analyzed by qPCR. p<0.05 when Siah2 expressing vector is compared to empty vector (EV). (C) Siah1a/2 is required for ATF4 protein expression. WT and *Siah1a^−/−^::Siah2^−/−^* MEFs and primary keratinocytes from WT and *Siah1a^+/−^::Siah2^−/−^* mice were treated with TM for 6 h (1 µg/ml). Cell lysates were prepared and the level of ATF4 protein was detected by immunoblots with the indicated antibodies. β-Actin served as the loading control. p<0.05 when Siah2 expressing vector is compared to EV. (D) Level of Siah2 transcripts was monitored following infection of either EV or Siah2 transcripts to WT or KO ATF4 MEFs maintained for 24 h under normoxic and hypoxic (H) conditions. (E–F) Siah2 expression increases ATF4 transcriptional activity. WT and *Atf4^−/−^* MEFs were transfected either with empty- or Siah2- expressing vector and 24 h later were subjected to hypoxia (1% O_2_; 6 h). Expression of CHOP mRNA (E) and VEGFA mRNA (F) was analyzed by qPCR. (G) ATF4 transcriptional activity is attenuated in *Siah1a^−/−^::Siah2^−/−^* cells. WT and *Siah1a^−/−^::Siah2^−/−^* MEFs were treated with TM (2 µg/ml) for 6 h and ATF3 mRNA was analyzed by qPCR. ** p<0.005, * p<0.05 compared to EV (D–F) in the same condition or to Siah1a/2 WT in the same condition (student's t test). The Western blot experiments were repeated three times and the qPCR results are shown as the mean values ± S.E. of three independent experiments.

Independent studies demonstrated that the prolyl hydroxylases PHD1 and 3 negatively regulate the protein level and transcriptional activity of ATF4 [45], [53], [54]. We thus tested whether the regulation of Siah1/2 transcription by ATF4 constitutes a feed-forward loop in which Siah2 degradation of PHD1/3 directly increases the availability and transcriptional activity of ATF4. Given the greater effect of the UPR on Siah2 transcription, ATF4 protein and transcript levels were measured in cells transfected with Siah2. We found that ATF4 protein levels were increased in cells expressing ectopic Siah2 (Figure 5A), with minor changes in ATF4 transcript levels (Figure 5B). Notably, the UPR-induced increase in ATF4 protein levels was attenuated in *Siah1a^−/−^::Siah2^−/−^* MEFs and in primary keratinocytes from *Siah1a^+/−^::Siah2^−/−^* mice (Figure 5C), suggesting that Siah1/2 increases ATF4 protein expression in response to UPR. Ectopic expression of Siah2 (Figure 5D) also increased the transcription of the ATF4 target genes CHOP and VEGFA in an ATF4-dependent manner, as the Siah2 effect was largely abolished in *Atf4^−/−^* cells (Figures 5E,F). Whereas hypoxia did not induce a notable increase in the levels of CHOP mRNA, it induced VEGF mRNA, to a similar degree as observed upon Siah2 expression alone (Figures 5E,F), suggesting that the Siah2-ATF4 axis also contributes to the increase in VEGFA mRNA levels under hypoxia. Moreover, the elevated ATF3 mRNA levels following TM treatment were attenuated in *Siah1a^−/−^::Siah2^−/−^* MEFs compared with WT MEFs (Figure 5G). These data point to the contribution of Siah2 to ATF4-dependent transcription in the UPR, thereby constituting a feed forward mechanism for the UPR.

**Figure 6:**
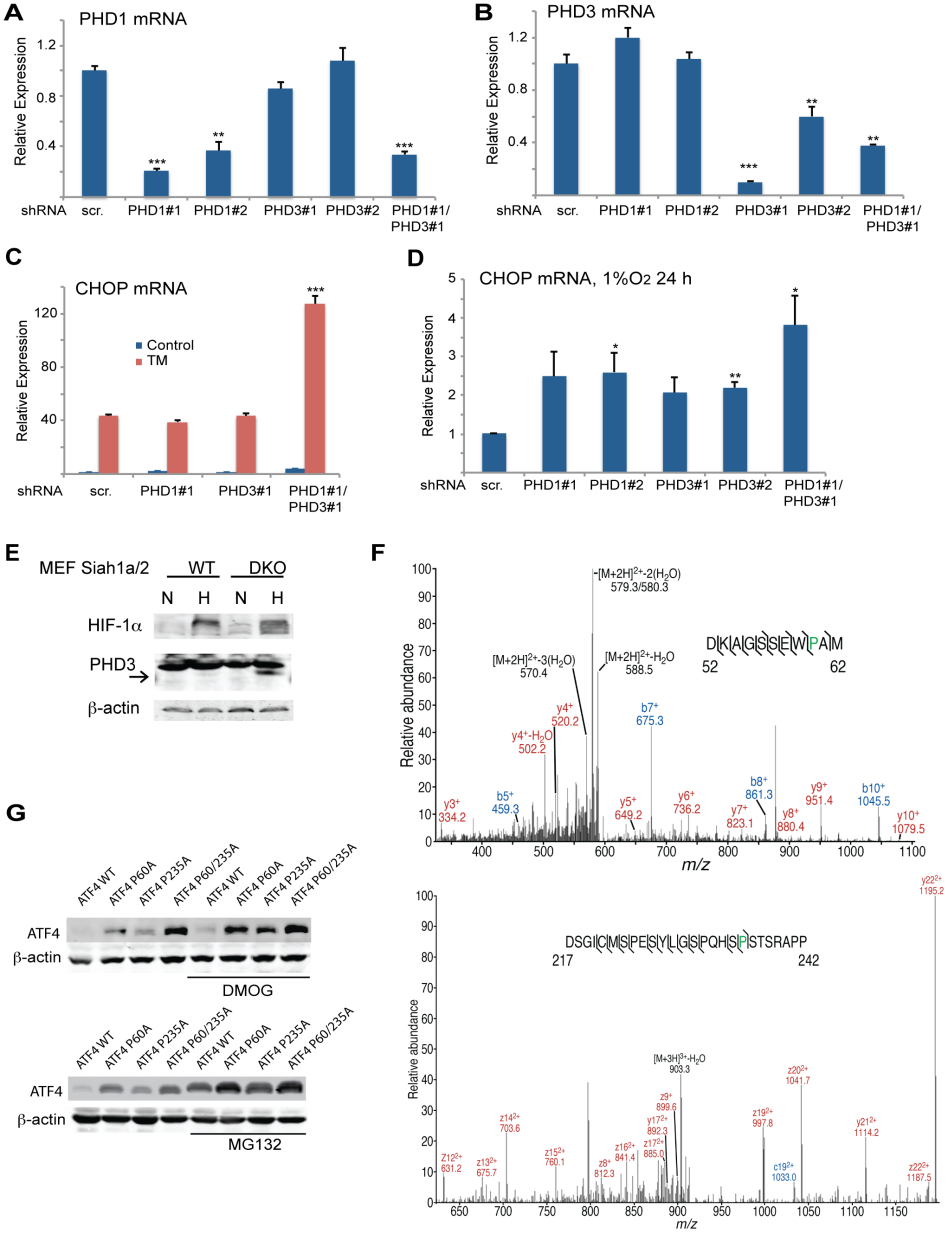
Prolyl hydroxylation of ATF4 on aa 60 and 235 by PHD1/3 limits ATF4 availability. (A–B) Specificity of shRNA used to KD PHD1/3. *Siah1a^−/−^::Siah2^−/−^* MEFs were infected with scrambled control or PHD1 shRNA and PHD3 shRNA alone or in combination, and treated with TM (1 µg/ml) for 6 h. The relative mRNA levels of PHD1 (A) and PHD3 (B) were determined by qPCR. The results are shown as the mean values ± S.E. of three independent experiments. Three independent shRNA were used to confirm the changes shown. (C) PHD1 and PHD3 cooperation is required and mediate the effect of Siah1a/2 on TM-induced CHOP transcription. *Siah1a^−/−^::Siah2^−/−^* MEFs were infected with different PHD1 shRNA or PHD3 shRNA, or their combination, or scrambled shRNA. Cells were treated with TM (1 µg/ml) and collected 6 h later. The relative transcription levels of CHOP were determined by qPCR. (D) PHD1 and PHD3 mediate the effect of Siah1a/2 on hypoxia-induced CHOP transcription. *Siah1a^−/−^::Siah2^−/−^* MEFs were infected with PHD1 shRNA and PHD3 shRNA alone or in combination, or control vector and exposed to 1% O_2_ for 6 h. The relative mRNA level of CHOP was determined by qPCR. (E) PHD3 protein is induced in *Siah1a^−/−^::Siah2^−/−^* cells. WT and *Siah1a^−/−^::Siah2^−/−^* MEFs were exposed to 1% O_2_ for 24 h prior to the analysis for the expression of HIF-1α, PHD3 and β-actin by Western blotting. The arrow points to the position of the endogenous PHD3 protein. (F) Annotated MS/MS spectra resulting in the identification of proline hydroxylation sites at P60 and P235. Identified fragment ions are shown, as are the detected sites of peptide backbone cleavage; *m*/*z*, mass to charge ratio. Note that site determining fragment ions resulted in localization of both sites of proline hydroxylation. (G) Mutations of the two identified proline hydroxylation sites at P235 and P60 to alanine stabilize ATF4 protein. 293T cells were transfected either with Flag-ATF4 or Flag-ATF4 presenting either a mutation at P60, P235, or both. After 24 h from transfection cells were treated overnight with vehicle, DMOG (0.5 mM; upper panel), or MG132 (5 µM; lower panel) followed by cell harvest and immunoblot analysis of ATF4 and β-actin. *** p<0.0005, ** p<0.005, * p<0.05 compared to ad shRNA scr. (A–D) in the same condition (student's t-test). The Western blot experiments were repeated three times and the qPCR results are shown as the mean values ± S.E. of three independent experiments.

Because PHD3 reduces ATF4 transcriptional activity [45], [53], [54], we next assessed whether the effect Siah2 elicited on ATF4 activity is PHD3-dependent. Therefore, we monitored changes in the levels of ATF4 target genes in *Siah1a^−/−^::Siah2^−/−^* MEFs transfected with PHD1 and PHD3 shRNA either alone or in combination. Expression of PHD1/3 shRNA in Siah1/2 DKO cells reduced PHD1/3 mRNA level by >50% (Figures 6A,B) while increasing the expression of CHOP mRNA following TM (Figure 6C) or hypoxia treatment (Figure 6D). Consistent with earlier reports [33], PHD3 protein levels increased in *Siah1a^−/−^::Siah2^−/−^* MEFs (Figure 6E). These findings confirm the role of Siah2, via its regulation of PHD3 stability, in the feed-forward regulation of ATF4.

We next determined whether PHD1/3 affects ATF4 by prolyl hydroxylation, similar to its control of HIF1α stability. ATF4, which enriched by immunoprecipitation from HEK293T cells that were transfected with both Flag-ATF4 and Myc-PHD3, was subjected to liquid chromatography tandem mass spectrometry (LC-MS/MS). This analysis identified two proline hydroxylation sites at P235 and P60 (Figure 6F), of which the P235 site is conserved among species (data not shown). ATF4 with proline mutated to alanine at each individual or both sites was assessed for altered expression level. Notably, mutation of either P60 or P235 increased the steady state levels of ATF4, with the double mutant revealing a more pronounced increase (Figure 6G). Further, inactivation of PHD (DMOG) or proteasomes (MG132) further increased the expression levels of ATF4, suggesting that additional factors may contribute to its overall expression (both transcriptionally and post translationally).

### ATF4-Siah2 is required for cellular response to severe ER stress

**Figure 7:**
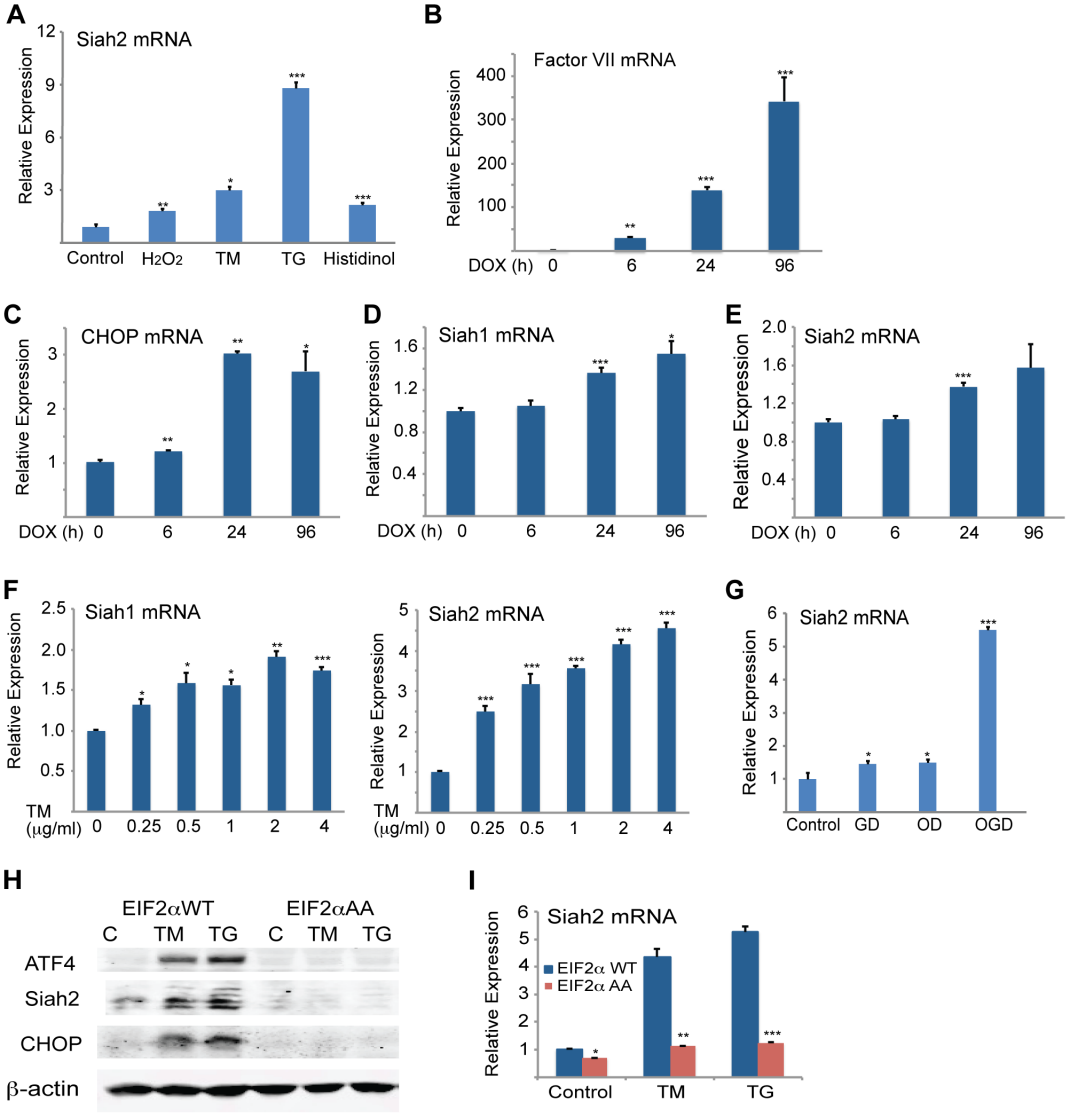
Severe ER stress conditions are required to induce Siah1/2 transcription by UPR. (A) Various forms of stress increase Siah2 mRNA levels. MEFs were treated with H_2_O_2_ (50 µM), TM (1 µg/ml), TG (1 µM) or histidinol (2 µM) for 8 h and Siah2 mRNA was analyzed by qPCR. Relative expression shown represents Siah2 transcript level. (B–E) CHO cells were treated with doxycycline (100 ng/ml) (83) and cells were collected after 0, 6, 24, and 96 h. The relative mRNA level of Factor VII (B), CHOP (C), Siah1 (D) and Siah2 (E) were determined by qPCR. The results are shown as the means ± S.E. of three independent experiments. (F) MEF cells were treated with increasing amount of TM and after 3 h the relative mRNA levels of Siah1 and Siah2 were determined by qPCR. (G) Siah2 is induced upon oxygen and glucose deprivation. WT or *Siah1a^−/−^::Siah2^−/−^* MEFs were subjected to glucose derivation (GD), oxygen deprivation (1%O_2_; OD) or their combination (OGD) for 12 h followed by cell harvest and RNA analysis using qPCR for levels of Siah2 transcripts. (H–I) ER-stress induction of Siah2 protein and mRNA levels is attenuated in eIF2α AA MEFs. Littermate-matched MEFs of the indicated genotypes were subjected to treatment with TM (1 µg/ml) or TG (1 µM). Cell lysates were prepared 8 h later and levels of ATF4, Siah2 and CHOP proteins were detected by immunoblots. β-actin served as the loading control (H). RNA was prepared 6 h after treatment with TM or TG and was used for qPCR to quantify the levels of Siah2 transcript, relative to levels of H3.3A mRNA (I). *** p<0.0005, ** p<0.005, * p<0.05 compared to control (panels A–G) * p<0.08 compared to WT (panels H–I) (student's t-test). The Western blot experiments were repeated three times and the qPCR results are shown as the mean values ± S.E. of at least three independent experiments.

To determine the significance of Siah activation by- and its augmentation of- the UPR we monitored the changes in Siah activation and its possible contribution to different forms of ER stress. Oxidative and ER stresses both effectively induced Siah2 mRNA levels, with TG and histidinol eliciting the greatest and the least effects, respectively (Figure 7A). To determine the conditions required for Siah activation by the UPR, we monitored the degree of its transcriptional activation following different levels of ER stress. Relatively mild levels of ER stress, which are elicited upon the inducible expression of coagulation factor VII [55] (Figure 7B), increased the transcript levels of CHOP (Figure 7C) and ATF3 (not shown), but not of Siah1 (Figure 7D) or Siah2 transcripts (Figure 7E). Similarly, the treatment with increasing doses of TM revealed that only concentrations in excess of 0.25 µg/ml resulted in notable activation of Siah1/2 transcription (Figure 7F). These observations suggest that low levels of ER stress do not engage Siah1/2 in the ER stress response. Conversely, exposure to severe ER stress conditions resulted in marked increase of Siah1/2 transcription.

Severe ER stress is often seen under conditions of glucose and oxygen deprivation, as occurs in cachexia, ischemia, or tumorigenesis [56]–[62]. Therefore, we examined the effects of oxygen and glucose deprivation on Siah2 and ATF4 expression. While there was limited change in Siah2 or ATF4 mRNA levels in cells deprived of glucose alone, deprivation of both glucose and oxygen resulted in a marked increase (6-fold) in Siah2 mRNA levels (Figure 7G).

To further substantiate the link between Siah and the UPR we assessed possible changes in Siah2 activation in cells with homozygous Ser51Ala knock-in mutation at the phosphorylation site in eIF2α, a key component in the PERK–ATF4 pathway. Phosphorylation mutant of eIF2α abolishes ATF4 synthesis, while causing severe ER stress due the inability to attenuate protein synthesis and the lack of compensatory mechanisms activated by ATF4. Thus, we assessed whether activation of Siah2 following UPR would mirror that of ATF4 and whether this activation is dependent on the phosphorylation of eIF2α. Strikingly, Siah2 expression at both the Siah2 protein (Figure 7H) and RNA levels (Figure 7I) phenocopied that of ATF4, as it was no longer observed following ER stress in the phosphomutant eIF2α cells. These findings further substantiate the integral role of eIF2α phosphorylation-dependent ATF4 synthesis with a concomitant effect on Siah2 transcription and contribution to the UPR.

**Figure 8:**
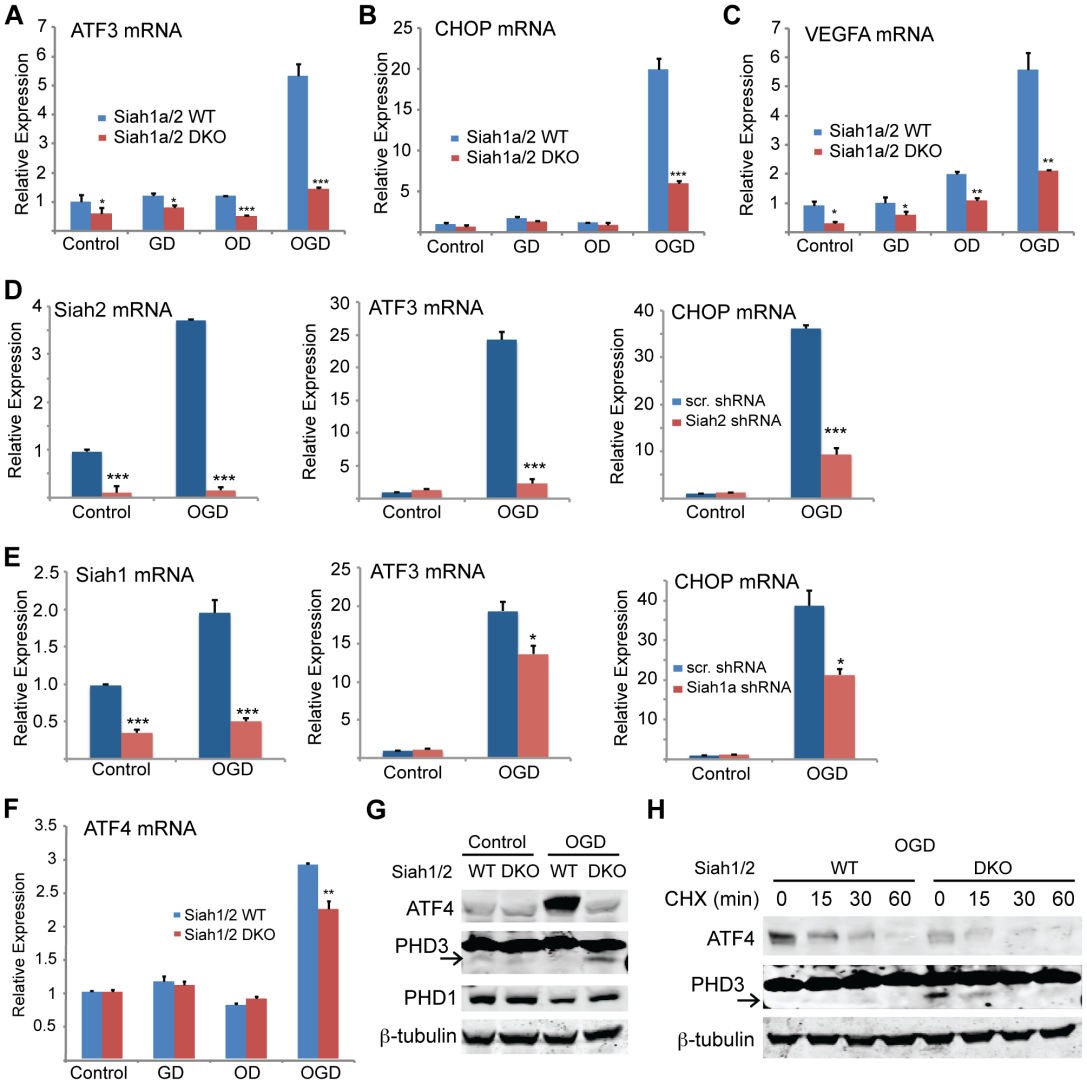
ATF4 transcriptional activity following oxygen glucose deprivation is Siah1/2 dependent. (A–C) ATF4 transcriptional activity under oxygen and glucose deprivation is Siah2 dependent. WT or *Siah1a^−/−^::Siah2^−/−^* MEFs were subjected to glucose deprivation (GD) oxygen deprivation (1% O_2_; OD) or their combination (OGD) for 12 h followed by cell harvest and RNA analyses. qPCR analysis was performed for ATF3 (A), CHOP (B) and VEGFA (C) using qPCR. (D–E) MEF cells were either infected with scramble shRNA, Siah2 shRNA (D) or Siah1 shRNA (E) and subjected to glucose deprivation and 1% O_2_ (OGD) for 12 h followed by cell harvest and RNA analysis using qPCR to determine mRNA levels Siah2, ATF3, and CHOP. (F) WT or *Siah1a^−/−^::Siah2^−/−^* MEF were subjected to glucose deprivation (GD), oxygen deprivation (1% O_2_; OD) or their combination (OGD) for 12 h followed by cell harvest and RNA analysis using qPCR for ATF4 mRNA levels. (G) WT and *Siah1a^−/−^::Siah2^−/−^* MEFs were exposed to glucose deprivation and 1% O_2_ (OGD) for 12 h prior to the analysis for the expression of ATF4, PHD1, PHD3 and β-tubulin by Western blotting. Arrow points to the position of the endogenous PHD3 protein. (H) WT and *Siah1a^−/−^::Siah2^−/−^* MEFs were subjected to glucose deprivation and 1% O_2_ (OGD) for 12 h prior to the treatment with the protein synthesis inhibitor cycloheximide (40 µg/ml) for 15, 30, and 60 minutes. Cell lysates were subjected to Western blotting using ATF4 and PHD3 antibodies. β-tubulin served as the loading control. Arrow points to the position of the endogenous PHD3 protein. *** p<0.0005, ** p<0.005, * p<0.05 compared to Siah1a/2 WT (A–C, F) or scr. shRNA (D–E) in the same condition (student's t-test). The Western blot experiments were repeated three times and the qPCR results are shown as the mean values ± S.E. of three independent experiments.

**Figure 9:**
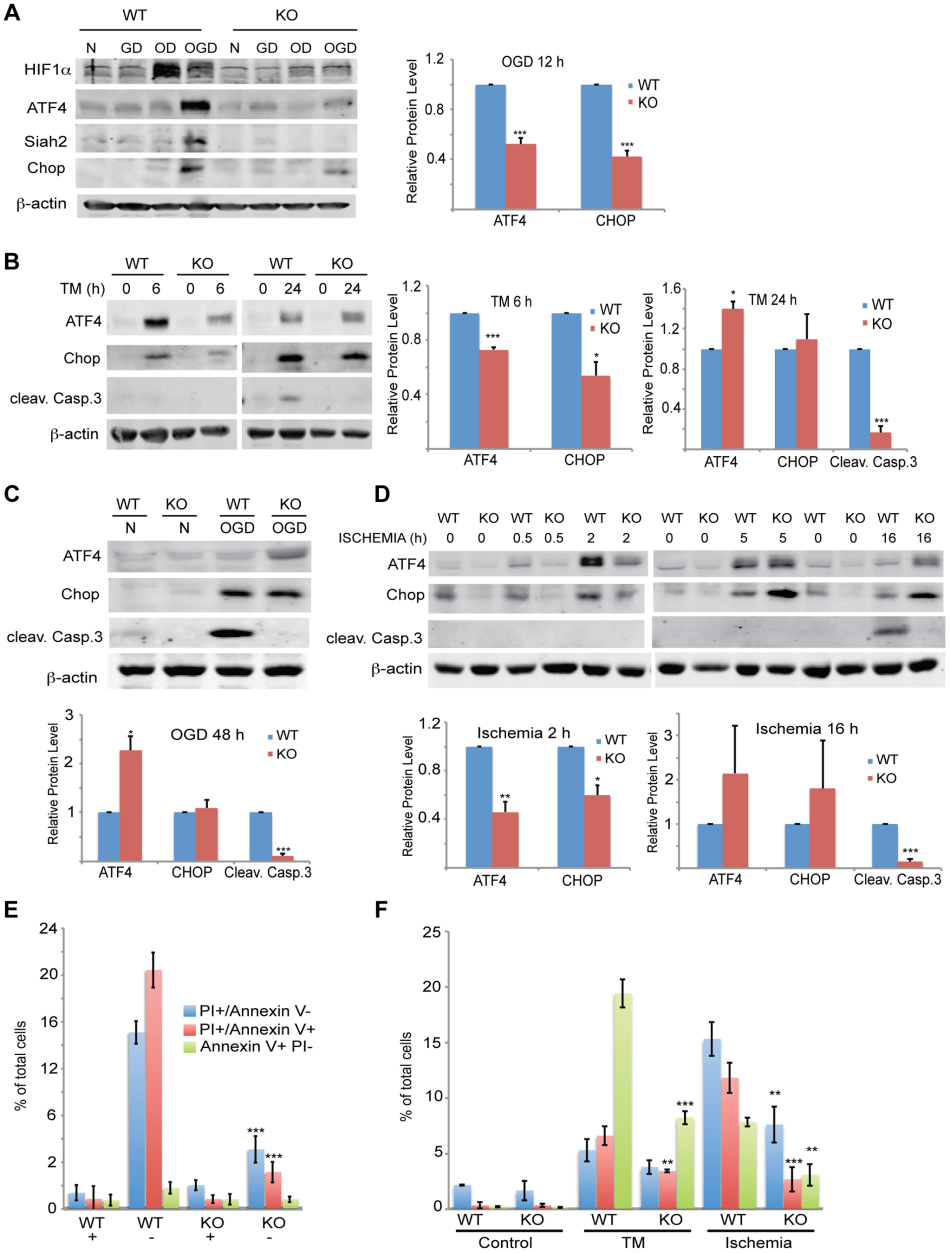
Siah1/2 activation following severe ER stress is required for UPR-induced cell death program. (A) Siah2 is required for ATF4 protein expression under oxygen and glucose deprivation. WT and *Siah1a^−/−^::Siah2^−/−^* MEFs were subjected to normoxia in rich media (N), glucose deprivation (GD), oxygen deprivation (OD) alone or in combination (OGD), for 12 h prior to the cell harvest and immunoblot analysis of HIF-1α, ATF4, Siah2, CHOP and β-actin. To the right is shown the quantification from three independent experiments of the normalized values. (B) WT or *Siah1a^−/−^::Siah2^−/−^* MEF were subjected to TM treatment for the indicated time periods, followed by cell harvest and Western blot analysis using the indicated antibodies. To the right is shown the quantification from three independent experiments of the normalized values. (C) WT or *Siah1a^−/−^::Siah2^−/−^* MEF were subjected to normal culture conditions (N) or oxygen+glucose deprivation (OGD) for 48 h followed by cell harvest and Western blot analysis with the indicated antibodies. Quantification of the normalized values based on three independent experiments is shown below the immunoblot. (D) WT or *Siah1a^−/−^::Siah2^−/−^* MEF were subjected to ischemia conditions (1% O_2_ without nutrients) for the indicated time periods, followed by cell harvest and immunoblot analysis with the indicated antibodies. Quantification of the normalized values based on three independent experiments is shown below the immunoblot (E) Siah1a/2 are required for OGD-induced cell death. Histogram showing the % of total cells exhibiting PI+/Annexin V-, PI+/Annexin V+, and PI+/Annexin V+ uptake as indicated by FACS analysis. The data shown represent experiments performed in triplicate (n = 10,000 cells per replicate). (F) Siah1/2 are required for TM- and ischemia-induced cell death. Experiment was performed as indicated in panel E except that MEFs were subjected to control, TM (1 µg/ml for 24 h) treatment, or ischemic (no oxygen and nutrients for 16 h) conditions. *** p<0.0005, ** p<0.005 compared to WT in the same condition (student's t test). The Western blot experiments were repeated three times and the qPCR results are shown as the mean values ± S.E. of three independent experiments.

The induction of Siah2 by glucose/oxygen deprivation prompted us to determine the effects of these conditions on ATF4 target gene expression. Notably, the mRNA levels of ATF3, CHOP, and VEGFA were markedly increased under conditions of glucose and oxygen deprivation (4- to 6-fold). Significantly, this effect was Siah1/2-dependent because the increase was essentially abrogated in *Siah1/2* DKO cells (Figures 8A–C), consistent with attenuated expression of ATF4 protein (Figure 9A). The contribution of Siah2 (Figure 8D) was more pronounced as compared with that of Siah1 (Figure 8E), consistent with our earlier experiments. There were marginal differences in ATF4 mRNA expression under the same experimental conditions between *Siah1a^+/+^:Siah2^+/+^* and *Siah1a^−/−^:Siah2^−/−^* cells (Figure 8F).

To determine the effects of Siah1/2 on PHD1 and PHD3 protein levels during UPR, WT and *Siah1a^−/−^::Siah2^−/−^* (DKO) MEFs were subjected to glucose and oxygen deprivation. Notably, glucose and oxygen deprivation caused increase in PHD3 protein levels in *Siah1/2* DKO MEFs, but not in WT MEFs. Further, PHD1 protein level decreased in WT, but not in *Siah1/2* DKO MEFs subjected to OGD (Figure 8G). Cycloheximide chase analysis allowed us to follow changes in the half-life of the PHD3 protein in *Siah1/2* WT and DKO MEFs that were subjected to OGD. The short half-life of PHD3 was detectable in *Siah1/2* DKO MEFs (at the 15 minute time point), but not in the WT MEFs (Figure 8H). Correspondingly, ATF4 half-life was also shorter in the *Siah1/2* DKO MEFs, following ODG treatment (Figure 8H). These finding substantiate the role of Siah1/2 in determining PHD3 as well as ATF4 stability, following OGD.

Given the Siah2-dependent expression of ATF4 in response to the UPR, we monitored changes in both ATF4 protein and ATF4 target gene CHOP levels, as well as in the degree of apoptosis following different levels of ER stress. The Siah2-dependent increase in ATF4, as well as in CHOP and HIF1α protein levels was enhanced in response to glucose/oxygen deprivation (Figure 9A). This increase was notably blunted in the Siah1/2 DKO MEFs. HIF1α expression levels were reduced during OGD, compared with hypoxia alone, (Figure 9A), consistent with the inhibition of HIF1α translation during severe ER stress conditions [63], [64]. Time course analysis following TM treatment revealed that ATF4 and its target gene CHOP were upregulated to a lesser degree in the Siah2 KO MEFs (Figure 9B) at the 6 h time point. Whereas the degree of ATF4 activation was reduced 24 h after TM treatment, the level of CHOP expression was comparable, and the expression of the apoptotic marker cleaved Caspase 3 were increased. Notably, although CHOP and ATF4 expression were still observed in the Siah1/2 DKO MEFs, Caspase 3 was not activated in these cells (Figure 9B). Additionally, the expression of ATF4, and its target gene CHOP, was Siah-dependent at an early time point following exposure to ER stress stimuli of increasing severity, represented here by TM (6 h; Figure 9B), the more severe OGD (8 h; Figure 9A) or the most severe ischemia-like condition (0.5, 2 h; ODG+ lack of nutrients; Figure 9D). Moreover, at these early time points there was no evidence for Caspase 3 cleavage or the induction of apoptosis. Only at later time points, including 24 h after TM, 48 h after OGD (Figures 9B,C), or 16 h after ischemic conditions (Figure 9d), was cleaved Caspase 3 observed in the WT but not the Siah1/2 DKO cells, indicating that Siah1/2 are required for UPR-induced cell death (Figures 9B–D, respectively). Of interest, at these later time points, the level of ATF4 was increased in the Siah1/2 DKO cells, (Figures 9B–D), suggesting that ATF4 facilitates survival rather than death programs in the absence of Siah1/2. Consistent with the finding that cell death following UPR requires Siah1/2, flow cytometric analyses confirmed that Siah1/2 DKO cells were significantly more protected from early and advanced cell death, as indicated by PI or Annexin+PI staining (Figures 9E,F).

Analysis performed at a later time point (48 h) revealed that the Siah1/2 DKO cells were protected from OGD induced apoptosis, reflected by the levels of PI and Annexin V uptake (Figure 9E). Similarly, the exposure of cells to ischemic conditions resulted in a lower degree of cell death in the Siah1/2 DKO cells, at the early time points (16/24 h) (Figure 9F). These findings establish the importance of Siah2 in conferring cell commitment to apoptosis in response to severe ER stress conditions.

**Figure 10:**
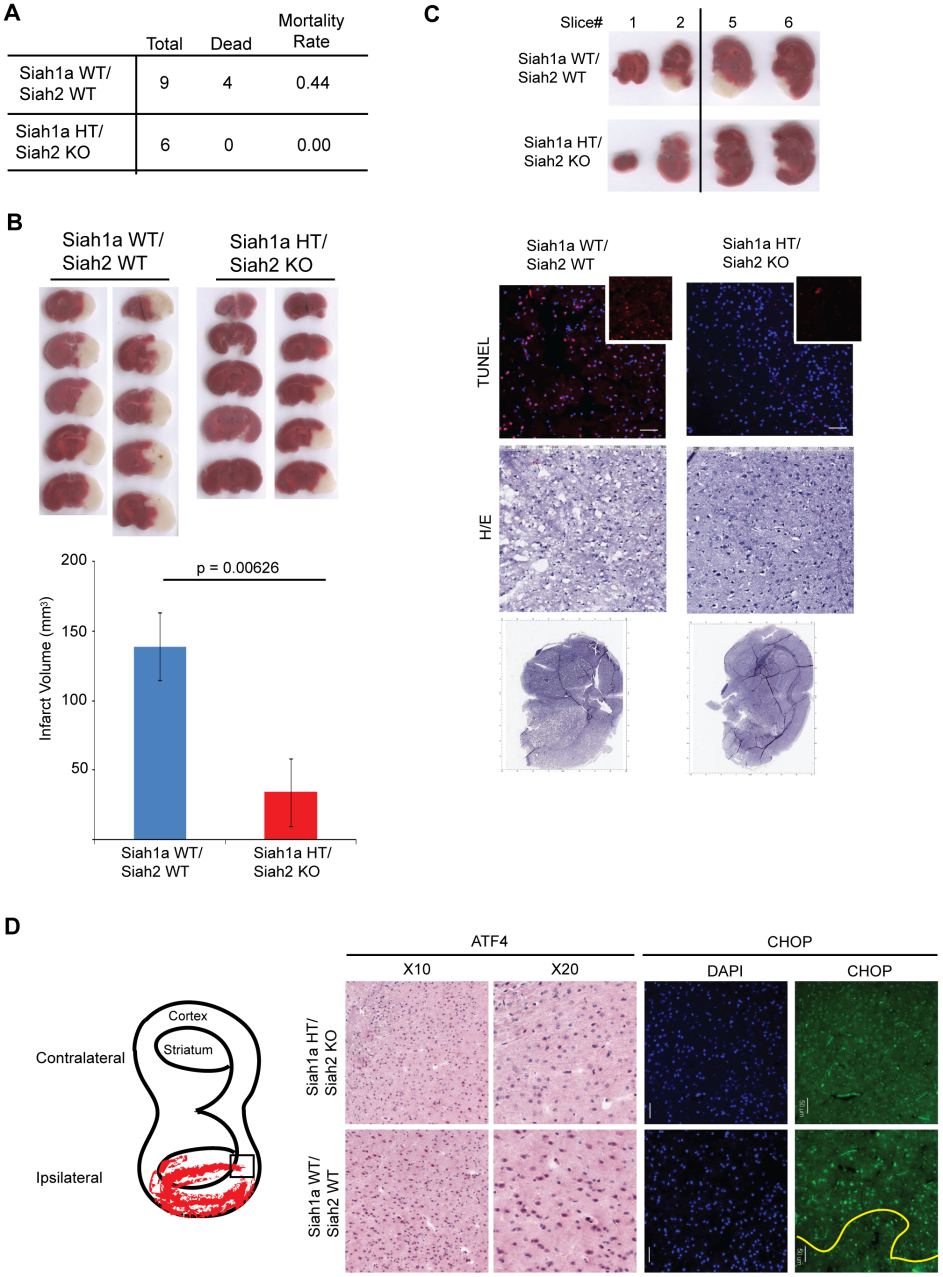
Siah1/2 mutant animals are protected from neuronal ischemia-induced cell death. (A) The mortality rate was calculated as the number of dead mice at 24 h after induction of MCAO in *Siah1a^+/+^::Siah2^+/+^* and *Siah1a^+/−^::Siah2^−/−^* mice. HT = heterozygous genotype of the Siah1a animals. (B) Brain sections from *Siah1a^+/+^::Siah2^+/+^* and *Siah1a^+/−^::Siah2^−/−^* mice were stained with Triphenyltetrazolium chloride (TTC) at 24 h after direct MCAO. The infarct volumes of brain sections were calculated as described in the Methods section. Five representative sections from two different mice of each group are shown. (C) At 24 h after MCAO, the brain sections were subjected to fixation, sectioned and further stained with H&E and ApopTag (TUNEL). Magnified TUNEL signal (red) is shown in the insets. The adjacent sections (#1, #2, #5 and #6) were stained with TTC solution. The scale bars indicate 50 µm. (D) Tissue sections adjacent to the ischemic core areas were analyzed by immunohistochemistry using the indicated antibodies. The square inset in the cartoon marks the area from which images were taken. The ischemic core was localized below the yellow line.

To further determine the role of Siah1/2 in cell death induced by severe ER stress such as ischemia, we used the middle cerebral artery occlusion model (MCAO), a surgical model of cerebral ischemia known to induce severe ER stress and cell death [65], [66]. Notably, 44% of the WT but none of the *Siah1a^+/−^::Siah2^−/−^* mice died within 24 h following permanent MCAO, indicating that WT mice are more sensitive to brain ischemia compared with the *Siah1a^+/−^::Siah2^−/−^* mice (Figure 10A). Consistently, the infarct volumes of WT mice were higher, compared to those observed in the *Siah1a^+/−^::Siah2^−/−^* mice [mean of 138.84 vs. 34.13 mm^3^] (Figure 10B). TUNEL staining of the most affected slices (slices #3 and #4 from the anterior side) revealed the presence of TUNEL-positive dead cells within the ischemic area of WT animal-derived brains (Figure 10C). Consistent with independent findings indicating that increased infarct size results in more pronounced cell death in WT brain under ischemia, ATF4 and its downstream gene, CHOP, were highly expressed in areas adjacent to the ischemic cores of brains from WT mice but not within equivalent areas of the brains of the *Siah1a^+/−^::Siah2^−/−^* that were subjected to the same procedure (Figure 10D). Our analysis was confined to the adjacent areas due to extensive cell death within the ischemic core regions; those regions have been previously reported to express reduced level of ER stress-related proteins (58). Taken together, these results establish the role of Siah in the fine-tuning of ER stress during brain ischemia, as reflected by the degree of protection from cell death seen in the *Siah1a^+/−^::Siah2^−/−^* mice.

## Discussion

The UPR constitutes a tightly controlled signaling network that dictates the ability of a cell to cope with stress and ultimately determines its fate. This well-orchestrated network is primarily regulated by three ER-anchored sensors, IRE1α, PERK, and ATF6α, which engage in crosstalk and feedback mechanisms to drive distinct cellular response programs. In spite of our current understanding of UPR and related pathways, mechanisms underlying cell decision to undergo adaptation versus death programs, in response to UPR of differing magnitudes, has been poorly understood.

Here, we demonstrate that the Siah1/2 ubiquitin ligases are an integral part of the UPR, as they are (i) activated by UPR transducers ATF4 and sXBP1, (ii) contribute to the degree of ATF4 transcriptional activation thereby increasing the ATF4 output, and (iii) primarily mediate the severe UPR stimuli by conferring cell death programs. Our experiments suggest that Siah1/2 ubiquitin ligases constitute an obligatory fine-tuning mechanism that favor cell death under severe UPR conditions.

The role of UPR in the regulation of Siah1/2 transcription constitutes an important addition to the previously established link between DNA damage ability to activate Siah1 (but not Siah2) through p53 binding to the Siah1 promoter [39]–[42]. As p53 is also induced by UPR and the greater ER stress program [67], our findings offer important insight into the role of diverse ER stress-inducible programs, namely, ATF4, sXPB1 and p53 in the activation of Siah1/2 genes.

In this newly identified regulatory axis, Siah2 is activated by two of the three UPR signaling pathways, PERK–ATF4 and IRE1α–sXBP1, in response to a mild to severe but not low level of ER stress. Thus, a threshold mechanism exists for ATF4 or sXBP1 induction of Siah2 mRNA, potentially upon cooperation with additional transcription factors, or following select post-translational modifications occurring following more severe UPR conditions. Once activated, Siah1/2 ligases attenuate the level of PHD3-dependent prolyl-hydroxylation of ATF4, which includes proline residues 60 and 235. Indeed, ATF4, which is mutated at these residues, is more stable enabling greater activation of downstream target genes. Correspondingly, the overexpression of Siah increases, whereas the inhibition of Siah reduces, the levels of ATF4 in a PHD3-dependent manner. Thus, the activation of Siah1/2 augments the expression and activity of ATF4, thereby increasing the overall level of the UPR.

Our findings also clarify a long-standing debate underlying the mechanism of PHD1/3 regulation of ATF4 availability and activity. Whereas one report points to the importance of PHD3 catalytic activity in the control of ATF4 [45], another suggests that the catalytic activity may not be required for ATF4 regulation [53], pointing to the possible recruitment of additional factors that contribute to ATF4 stability and activity. Here, we identified two previously unknown sites on ATF4 that are subject to prolyl hydroxylation by PHD1/3 and demonstrate the role of this hydroxylation in the Siah2-dependent stabilization of ATF4. Important to note that we identified the prolyl-hydroxylation sites on ATF4 in vivo, as oppose to earlier attempts, that were carried out in vitro. The latter suggest that additional post-translational modifications may be required for PHD3-hydroxylation of ATF4. Previous reports identified the SCF complex component βTrCP as an ATF4 ubiquitin ligase. Since βTrCP requires ATF4 phosphorylation for its association and ubiquitination, it is of interest to determine the relationship between hydroxylation and phosphorylation of ATF4, particularly as the P235 site we identified is positioned within the phosphorylation sites mapped for recognition by βTrCP.

The significance of the Siah ubiquitin ligases to the overall UPR is reflected in their impact on cellular commitment to undergo death. Severe forms of ER stress, including exposure to higher levels of TM, OGD, or ischemia (where nutrients are also absent), results in cell death programs, which were markedly attenuated in the Siah1/2 KO cells. The evidence for the requirement of Siah1/2 for cell death programs was substantiated by biochemical (absence of cleaved caspase 3) and cellular (FACS analysis) read-outs, and more so, by using the genetic *Siah1^+/−^*::*Siah2^−/−^* animals. Ischemic brain injury was reported to occur in response to ER stress conditions, in an ATF4-dependent manner [65], [66]. Notably, ischemic brain injury was attenuated in the *Siah1^+/−^*::*Siah2^−/−^* animals, providing further evidence for the requirement of Siah2 in fine tuning of ER stress/ATF4-dependent cell death programs.

How does Siah contribute to cell death programs in response to ER stress? In the presence of Siah2, a higher level and activity of ATF4/CHOP induces cell death programs (PUMA, BAX). Under such conditions Siah2 also contributes to an increased rate of mitochondrial fission, as demonstrated in our earlier studies [28]. In the absence of Siah2, the expression of ATF4/CHOP is insufficient to induce the death signaling pathways, and instead promotes survival pathways (i.e., autophagy). Further, in the absence of Siah2 the rate of mitochondrial fission is reduced, contributing to protective mechanisms [28]. The latter is consistent with the finding that ATF4 is required for mitochondrial dynamics [68]. Correspondingly, Siah2 control of ATF4 expression may explain changes that underlie the switch from cell survival to death programs, under severe ER stress conditions. These findings highlight a mechanism underlying the fine-tuning of the UPR, addressing a long sought quest for mechanisms that distinguish the role of ER stress in recovery of normal homeostasis versus the activation of cell death.

Our studies also offer an initial link between UPR and hypoxia pathways. Through its common substrate, PHD3, Siah1/2 regulates key components in hypoxia (HIF1α) and UPR (ATF4). Noteworthy is that the cross talk among the different ER stress sensors may further augment the mechanism described in our studies. Namely, upregulation of Siah2 mRNA by sXbp1 points to a mechanism by which the IRE1 sensor may augment the activity of the PERK sensor. Conditions under which IRE1/sXBP1 are activated will induce Siah1/2, which in turn will augment ATF4 availability. Of interest is to note that the sXBP1 binding site on the Siah2 promoter overlaps with the hypoxia response element (HRE), raising the possibility that stress (ER or oxidative) that is sufficient to trigger either ATF4 or sXbp1 transcription may constitute the initial signal for Siah2 transcription. Subsequently, Siah1/2 stabilization of HIF1α could replace sXBP1 on Siah1/2 promoter, to sustain Siah1/2 mRNA levels. Alternatively, sXBP1 and HIF1α may cooperate on promoters where their response elements overlap, as seen for Siah1/2 promoters. Thus, Siah1/2 ligases contribute to key cellular pathways, hypoxia and the UPR, while being regulated by their primary components as part of a feed forward loop mechanism.

We showed that under conditions of glucose/oxygen deprivation, or ischemia, Siah1/2 ligases are required for ER stress/ATF4 activation of cell death programs. In agreement, the increased expression of Siah1 coincided with decreased PHD3 expression following cerebral ischemia [69]. Moreover, we expect that the role of Siah ligases in fine-tuning the UPR would be relevant to an array of pathological conditions associated with neuronal ischemia, fatty liver, and heart diseases [70]–[74].

Overall, our studies identify Siah2 as an important regulatory component of the UPR, which serves to fine-tune the degree of UPR, and in doing so, to impact the outcome of the UPR ability to initiate cell death programs.

## Materials and Methods

### Ethics statement

All animal work has been conducted according to relevant national and international guidelines in accordance with recommendations of the Weatherall report and approved. Our study protocols using *Siah1a^+/+^::Siah2^+/+^* and *Siah1a^+/−^::Siah2^−/−^* mice (129/BL6 background) were generated as previously described [75] and approved by the Institutional Animal Care and Use Committee (IACUC) of the Sanford-Burnham Medical Research Institute.

### Cell lines

HEK293T, CHO HeLa, Lu1205, and HEK 293T cells were maintained in Dulbecco's modified Eagle's medium (DMEM, Invitrogen) with 10% fetal bovine serum (FBS; Sigma) and 1% penicillin–streptomycin (Invitrogen) at 37°C. The melanoma cell line Lu1205 was a kind gift from Meenhard Herlyn. Siah1a/2 WT, Siah1a/2 KO, IRE1α WT, IRE1α KO, ATF4 WT, and ATF4 KO MEFs [10], [75], [76] were similarly maintained with the addition of 55 µM β-mercaptoethanol (Invitrogen) and 1× nonessential amino acids (Invitrogen). Cells were seeded and cultured to ∼60–80% confluency. On the day of the experiment, cells were treated with vehicle (DMSO), or 1–2 µg/ml tunicamycin (Sigma) in DMSO, or 1 µM of thapsigargin (Sigma) in DMSO, or subjected to hypoxia (1% O_2_). Glucose deprivation was performed by incubating the cells in complete DMEM or in DMEM lacking glucose (Invitrogen) and containing 10% dialyzed FBS (Invitrogen).

### Keratinocyte culture

Primary mouse keratinocytes were isolated from newborn mouse epidermis as described [77] and seeded at a density of 5×10^6^ cells per 60-mm dish (or equivalent concentrations) in a defined serum-free keratinocyte specific medium (Invitrogen). Doxycycline was purchased from Sigma.

### Antibodies and reagents

Antibodies to Siah2, GRP94, ATF4, XBP1, AKAP121, myc, β-actin, CHOP were from Santa Cruz Biotechnology. Anti-HIF-1α was a generous gift from Dr. Robert Abraham. Anti-Flag and anti-Siah1 were from Sigma, while anti-PHD1 and anti-PHD3 were from Abcam. All antibodies were used according to the suppliers' recommendations. MG132 was from Calbiochem. DMOG was from Sigma. Immunohistochemical analysis of ATF4 expression was performed using anti-ATF4 antibody from Abcam.

### LC-MS/MS

Samples were prepared from ATF4 immunoprecipitates (antibodies described above) using standard procedures. Briefly, protein samples were reduced, alkylated, trypsin-digested, desalted and dried in a speed vac. Samples were re-suspended in 0.1% formic acid/5.0% acetonitrile and analyzed using a MS2 HPLC, HTC-PAL autosampler, Captive Spray Source (Bruker-Michrom) and a LTQ Orbitrap Velos Pro mass spectrometer equipped with electron transfer dissociation (Thermo Fisher Scientific; [78]). Database searches were as described previously [78] against a mouse protein database (mouse.ipi.3.73) with the exception that proline hydroxylation (+15.99491 Daltons) was included as a differential modification. The accuracy of the proline hydroxylation site identifications was manually confirmed (Figure 6F).

### Statistical analysis

The data were analyzed by Student's -test. p<0.05 was considered statistically significant. To verify that the number of mice used in the study was sufficient to provide the statistical power we calculated the sample size using G*Power 3.1 software for power analysis, further aided by using the t-test for assuring sufficient difference between two independent groups.

### Siah2 promoter fragments

The human Siah2 promoter fragments were amplified from HeLa cell genomic DNA by PCR and cloned into the pGL3-luciferase reporter vector (Promega). All constructs were verified by DNA sequencing.

### Plasmids, cloning and mutagenesis

PHD3 tagged with myc, OGDH and Siah2 tagged with Flag were cloned as previously described [27], [33], [46], [79]–[81]. The luciferase reporter vector containing the ATF4 response element was constructed by cloning the promoter region of mouse *Trib3* gene (−2000 to +30 bp of transcription start site) into pGL3-luciferase. Mutagenesis of ATF4 and XBP1 binding site on the Siah2 promoter and mutagenesis of ATF4 on the hydroxylation sites were performed using QuickChange II XL mutagenesis kit (Invitrogen). The sites of mutation for ATF4 and XBP1 are respectively shown in small letters: 5′-GTCCGCGCGCGCCCCCGGGGCcTGtcTCAGCGGCTGTTCCAGAAG-3′ (ATF4), 5′-CGTGTCCAGGCGTattaTCCGCGCGCGCCCCCGG (XBP1). The primers used for mutagenesis on mouse ATF4 of the two proline in position 60 and 235 were the following: for the mutation of proline in position 60: 5′-GGCTCCTCGGAATGGgCGGCTATGGATGATGGCTT-3′ (in lower case is the mutated nucleotide); for the mutation of proline in position 235: 5′-TCTCCCCAGCATAGCgCCTCCACCTCCAGGGCCCCA-3′ (in lower case is the mutated nucleotide).

### Adenoviral infection

Adenoviruses expressing WT ATF4, and spliced XBP1 were previously reported [82], [83]. Adenoviruses-expressing green fluorescent protein or β-Gal were used as a control. MEFs were infected with adenoviruses at a multiplicity of infection of 20. Twenty hours after infection, the adenovirus was removed, cells were harvested and total RNA and protein were extracted for analysis.

### Gene silencing and transduction

The shRNA vectors for silencing of *Siah1a* or *Siah2* were previously described [28]. The sequences used for scramble, *Siah2* and *Siah1a* shRNA were respectively: caacaagatgaagagcaccaa (scramble), ccttggaatcaatgtcacgat (mSiah2), atttagctcaagtcgatatgc (mSiah1a). For silencing of the murine *PHD1 and PHD3* genes, shRNAs were from Sigma (TRCN0000273085, TRCN0000009744 TRCN0000009746). For transduction, viral particles were harvested after transfection of HEK293T cells with the plasmid of interest and matched packaging plasmids using Jet Prime (Polyplus Transfection). Target cells were infected with viral particles by inoculation in the presence of polybrene (4 µg/ml, Sigma). Stable clones were established by growing cells in media containing puromycin (1 µg/ml, InvivoGen). For the preparation of PHD3 shRNA #2, nucleotides corresponding to 357–375 nt of mouse *PHD3* coding sequence were synthesized and cloned into pSuper vector (pSup-PHD3) as previously described [33]. Empty pSuper vector was used as a control. RNAi expression vectors were introduced into MEFs by transfection (jetPrime,). After 48 h, cells were treated with or without hypoxia and harvested.

### Immunoprecipitation and western blotting

To extract whole cell lysate, cells were harvested using RIPA buffer (50 mM Tris-HCl pH 7.5, 150 mM NaCl, 1% Triton X-100, 0.1% SDS, 0.1% Na-deoxycholate, 1 mM EDTA, 1 mM sodium orthovanadate, 1 mM PMSF, 10 µg/ml aprotinin, and 10 µg/ml leupeptin). Cell lysates were subjected to SDS-PAGE and proteins transferred onto a nitrocellulose membrane (Osmonics Inc.). The membrane was probed with primary antibodies (described above) followed by a secondary antibody conjugated with fluorescent dye and detected using the Odyssey detecting system (LI-COR Bioscience).

### Hypoxia treatment

Cells were exposed to hypoxia (1% O_2_) in a hypoxia workstation (In Vivo 400; Ruskinn Corp.) and then processed immediately on ice.

### Luciferase assay

Cells in 24-well plates were transfected with 100 ng of Luciferase vector containing the Siah2 promoter region or the Luciferase vector containing the ATF4 response element and 80 ng of β-Gal vector using jetPrime in triplicate. After 24 h, cells were infected either with ATF4, spliced XBP1, or GFP virus for 24 h, prior to collection of cell lysate using 60 µl of reporter lysis buffer (Promega). Cell lysates (10 µl) were loaded onto a 96-well plate and luciferase activity was measured using a Veritas Microplate Luminometer (Turner Biosystems) according to manufacturers' instructions. β-Gal assay was used to normalize transfection efficiency. For the β-Gal assay, 10 µl of cell lysate was incubated in reaction buffer (100 mM NaH_2_PO_4_ pH 7.5, 0.1% O-Nitrophenyl-β-D galactopyranoside (ONPG), 1.2 mM MgCl_2_ and 50 mM β-mercaptoethanol) at 37°C for 20 min, and the reaction was stopped by addition of 1 M Na_2_CO_3_.The OD at 420 nm measured with a spectrophotometer was used to reflect β-Gal activity. The luciferase activity of each sample was divided by the β-Gal activity to calculate the relative luciferase activity.

### Chromatin immunoprecipitation (ChIP) assay

ChIP assays were performed using a Magna CHIPTM kit (Upstate). Triplicate biological samples were processed and analyzed. Briefly, cells (1.5×10^7^) were maintained in normoxia or hypoxia (1% O_2_) for 6 h, or treated with 1 µM of TG for 5 h, and crosslinked using 1% formaldehyde for 10 min at RT. The crosslinking was stopped by 5 M glycine. Cells were lysed and sonicated to obtain 200–500 bp chromatin fragments. Chromatin was immunoprecipitated with 5 µg of antibodies (Santa Cruz) and 20 µl of protein A/G magnetic beads in a total volume of 0.5 ml overnight at 4°C. After four washes, crosslinking of protein/DNA complex was reversed, DNA was purified using spin column, and subjected to qPCR analysis. The following primers corresponding to mouse Siah2 5′-untranslated region and spanning the HRE, ATF4 and XBP1 binding sites were as follows: 5′-GCGATCGACTCATTCAAGGGCTC-3′ and 5′-GCGTTCTGGTGCGCAGAG-3′.

### Microarray analysis

WT and Siah1a/Siah2 KO MEF cells were treated with TM or TG for 6 h, or subjected for 12 h to OD, GD or OGD, in duplicate. Total RNA (500 ng) was used for synthesis of biotin-labeled cRNA using an RNA amplification kit (Ambion). The biotinylated cRNA was labeled by incubation with streptavidin-Cy3 to generate probe for hybridization with the Mouse-6 Expression BeadChip (Illumina) that contain 48 K probes corresponding to mouse gene symbols. We analyzed the BeadChips using the manufacturers BeadArray Reader and collected primary data using the supplied Scanner software. Illumina BeadArray scanned data were pre-processed and normalized by Illumina GenomeStudio software (Illumina Inc, San Diego, CA). Probesets that are absent in all the study samples were removed from further analyses. To identify differentially expressed genes, the linear modeling approach and empirical Bayes statistics as implemented in the limma package [84] were employed. The Benjamini–Hochberg method was used to correct for the multiple comparison errors [85]. Principal component analysis (PCA) was performed with Partek Genomics Suite (Partek Inc. St. Louis, MO), and hierarchical clustering and other statistical analyses were performed using R/Bioconductor [86]. Genes with at least a 2-fold change at the 95% confidence level were considered as significant. The selected statistically significant genes in each of these experimental groups were than analyzed for functional enrichments using the Ingenuity Pathway Analysis (IPA) platform (Ingenuity Systems Inc., Redwood City, CA). The Gene Set Enrichment Analysis (GSEA) approach was also applied for the functional enrichment analyses [87]. A Fisher's exact test was employed to calculate the significant value, which determined the probability that an association between the genes within the dataset and the functional pathway could be explained by chance alone. Functional groups (or pathways) with a p-value<0.05 were considered to be statistically significant. The data obtained through our gene expression analysis has been deposited in the GEO public dataset (GSE39244).

### qRT-PCR analysis

Total RNA was extracted using a total RNA miniprep kit (Sigma) and digested with DNase I. cDNA was synthesized using oligo-dT and random hexamer primers for SYBR Green qPCR analysis. 18S rRNA and H3.3A were used as internal controls. Triplicate biological samples were used for the qPCR analysis. The PCR primers were designed using Primer3 and their specificity was checked using BLAST (NCBI). The PCR products were limited to 100–200 bp. The primers used for qPCR analysis were as follows:

mouse VEGFA forward 5′-ATCTTCAAGCCGTCCTGTGT-3′ and reverse 5′-GCATTCACATCTGCTGTGCT-3.′; mSiah2 forward 5′-GCTGAGAACTTTGCCTACAG-3′, and reverse 5′-GCTATGCCCAAATAACTTCC-3′; 18S rRNA forward 5′-GAGCGAAAGCATTTGCCAAG-3′, and reverse 5′-GGCATCGTTTATGGTCGGAA-3; mCHOP forward 5′-AAGCCTGGTATGAGGATCTGC-3′, and reverse 5′-GGGGATGAGATATAGGTGCCC-3′; mATF3 forward 5′-AGAGCTGAGATTCGCCATCC-3′, and reverse 5′-TGTTGACGGTAACTGACTCCA-3′;m spliced XBP1 forward 5′-CTGAGTCCGAATCAGGTGCAG-3′ (original CAG sequence was mutated to AAT to reduce the background signal from unspliced XBP-1) and reverse 5′-GTCCATGGGAAGATGTTCTGG-3′; mSiah1a forward 5′-GCTGAAAATTTTGCATATCG-3′ and reverse 5′-CCAGGAAAGTTTTAGGTTGG-3′; mSiah1b forward 5′-GAGATGAGCCGTCAGGCTGCTA-3′ and reverse 5′-GAATAGGTGGCAACACATAG; mATF4 FORWARD 5′-CCTGAACAGCGAAGTGTTGG-3′ and reverse 5′-TGGAGAACCCATGAGGTTTCAA-3′; mPHD1 forward 5′-AGTCCTTGGAGTCTAGCCGAG-3′ and reverse 5′-TGGCAGTGGTCGTAGTAGCA-3′; mPHD3 forward 5′-TTATGTTCGCCATGTGGACAA-3′ and reverse 5′-gcgtcccaattcttattcaggta-3′; hFactor VII forward 5′-AACCCCAAGGCCGAATTGT-3′ and reverse 5′-CGCGATCAGGTTCCTCCAG -3′. mMMP10 forward 5′- GAGCCACTAGCCATCCTGG, and reverse 5′- CTGAGCAAGATCCATGCTTGG-3′; mWNT5a forward 5′- CTCCTACAGTGTGGTTGTCAGG-3′, and reverse 5′- GCGCATCCATAAAGAGTCTTGA-3′; mWNT6 forward 5′- CTCCTACAGTGTGGTTGTCAGG-3′, and reverse 5′- GCGCATCCATAAAGAGTCTTGA-3′; mSOX11 forward 5′- CGAGCCTGTACGACGAAGTG-3′, and reverse 5′- AAGCTCAGGTCGAACATGAGG-3′; mPDGFC forward 5′- GCCAAAGAACGGGGACTCG-3′, and reverse 5′- AGTGACAACTCTCTCATGCCG-3′; mNRP1 forward 5′- ACCTCACATCTCCCGGTTACC-3′, and reverse 5′- AAGGTGCAATCTTCCCACAGA-3′; mALDH1 forward 5′- TTCCCACCGTCAACCCTTC-3′, and reverse 5′- CCAATCGGTACAACAGCCG-3′; mIGFBP6 forward 5′- TGCTAATGCTGTTGTTCGCTG-3′, and reverse 5′- CACGGTTGTCCCTCTCTCCT-3′; mGRB10 forward 5′- GGACAAATCGGAAGAGTGATCG, and reverse 5′- CATCCGTGTGCTCCCGTTAC-3′; mGPX7 forward 5′- TCCGAGCAGGACTTCTACGAC-3′, and reverse 5′- TCTCCCTGTTGGTGTCTGGTT-3′; mCYP7b1 forward 5′- GGAGCCACGACCCTAGATG-3′, and reverse 5′- GCCATGCCAAGATAAGGAAGC-3′; mCBR2 forward 5′- GGGCAGGGAAAGGGATTGG-3′, and reverse 5′- CCACACACACGGGCTCTATTC-3′; mIGF2 forward 5′- GTGCTGCATCGCTGCTTAC-3′, and reverse 5′- ACGTCCCTCTCGGACTTGG-3′; mPTGIS forward 5′- GCCAGCTTCCTTACCAGGATG-3′, and reverse 5′- GAGAACAGTGACGTATCTGCC-3′; mRab6 forward 5′- GGAGACTTCGGGAATCCGC-3′, and reverse 5′- ACTGTCATACATGAATCGGGTGA-3′; mGOLT1b forward 5′- ATGATCTCCCTCACGGATACG-3′, and reverse 5′- TCGAGACCAATTACAAAAGCCAA-3′; mHSPA5 forward 5′- ACTTGGGGACCACCTATTCCT, and reverse 5′- GTTGCCCTGATCGTTGGCTA-3′.

### Cerebral ischemia and assessment of infart volume and cell death analysis

*Siah1a^+/+^::Siah2^+/+^* and *Siah1a^+/−^::Siah2^−/−^* mice (male, 8–9 weeks old with 22–25 mg body weight) were subjected to induction of focal cerebral ischemia through intraluminal MCAO. Mice were anesthetized with 2.0–2.5% isoflurane administered by mask and MCAO was performed as described [88]. Briefly, unilateral MCAO was induced by insertion of silicone rubber-coated monofilament (Doccol Corp.). The silicone suture (size 6-0, diameter 0.09–0.11 mm) introduced from the common carotid artery was advanced into the internal carotid artery (10–11 mm from the common carotid artery bifurcation site). The filament was left in place for 24 h for pMACO. The post-ischemic mice were sacrificed, the brain was collected and placed at −20°C for 12 min. Then, coronal slices with 1 mm thickness were obtained by cutting using brain matrix. Sections were stained with 2% 2,3,5-triphenyltertrazolium chloride (TTC) solution for 25 min at 37°C. The sections were fixed with 10% neutral buffered formalin solution (Sigma-Aldrich) at 4°C.

Coronal brain slices were scanned with a high-resolution scanner (HP ScanJet G4010). The healthy non-ischemic area and infarct area were measured using Image J1.44 (NIH). To avoid an edema effect, infarct area was calculated by subtracting healthy area of ipsilateral hemisphere from total area of contralateral hemisphere as described [82]. Infarct volume was calculated by adding all brain sections with 1 mm thickness.

To verify that the number of mice used in the study is sufficient to provide the statistical power we calculated the sample size using G*Power 3.1 software for power analysis, which was further aided using the t-test to assure sufficient difference between two independent groups. To have a 95% probability of detecting a difference in means of 4.3 standard deviations, a sample size of three mice in each treatment group was calculated to be necessary. Accordingly the sample size used in these experiments is sufficient to support statistically significant conclusions. Of note, we used a larger group size for the WT animals since 44% of the WT mice died within 24 h following permanent MCAO. The increased group size for the WT enabled to secure the number of mice needed for statistical power.

### TUNEL staining

Brain slices from *Siah1a^+/+^::Siah2^+/+^* and *Siah1a^+/−^::Siah2^−/−^* mice were obtained after 24 h of pMCAO. Among the slices, #3 and #4 slices were further sectioned through paraffin-embedded method and stained using ApopTag-red (Millipore) according to manufacturer's direction. Immunohistochemistry analyses of adjacent slices were performed for expression of ATF4 and CHOP.

### Histological analyses

Tissue collected from *Siah1a^+/+^::Siah2^+/+^* and *Siah1a^+/−^::Siah2^−/−^* mice were fixed in Z-fix (buffered zinc formalin fixatives, Anatech) overnight. After fixation, tissues were washed twice with PBS and processed for paraffin embedding. Brain embedded in paraffin blocks were sliced at 5 µm and stained with hematoxylin and eosin. For ATF4 and CHOP staining, all cryosections were fixed with Z-fix (buffered zinc formalin fixatives, Anatech) and followed by a blacking of non-specific binding sites (Dako/Agilent Inc.) for 30 min at room temperature. Antigen retrieval was performed in a pressure cooker (Decloaking chamber, Biocare Medical) in citrate buffer (pH 6.0) and used for CHOP and ATF4 immunostaining. Antibodies/dilutions for the following markers were used in antibody diluent (Dako) overnight at 4°C. For CHOP analysis secondary antibody labeled with Alexa Fluor 488, was placed on tissue sections for 1 h at room temperature (1∶400, Molecular Probes). Nuclei were counterstained using *SlowFade* Gold Anti-fade reagent with 4′,6-diamidino-2-phenylindole (DAPI; Vector Laboratories). For immunohistochemistry analysis of ATF4 the staining was visualized using an alkaline phosphatase technique (Vector red alkaline substrate kit I, Vector Laboratories).

### Annexin V and propidium iodide staining and flow cytometric analyses

Siah1a/2 WT and double knockout MEFs were grown under control (normoxic, high glucose), OD (1% O_2_) and OGD (1% O_2_, glucose-free for 48 h), or ischemic condition (no nutrient media for 16 h). The cells were then harvested and immediately stained with Annexin V-FITC and propidium iodide using the Biovision Annexin V-FITC Apoptosis Kit (Biovision, USA; K101-100). The cells were subjected to FACS analysis using a FACSCanto (BD Biosciences, USA) and FlowJo software (TreeStar, USA) (n = 10,000 cells per replicate). The data shown represent experiments performed in triplicate.
